# Optimal timing for frozen-thawed embryo transfer: evidence from a systematic review and meta-analysis

**DOI:** 10.3389/fendo.2026.1851394

**Published:** 2026-07-15

**Authors:** Guangzhong Jiao, Huayu Lian, Xiaoyan Liu, Xunlan Yin

**Affiliations:** 1 Department of Reproductive Medicine, The Affiliated Yantai Yuhuangding Hospital of Qingdao University, Yantai, Shandong, China; 2 Shandong Provincial Key Medical and Health Laboratory of Reproductive Health and Genetics (Yantai Yuhuangding Hospital), Yantai, China; 3 Department of Food and Biological Engineering, Yantai Institute of Technology, Yantai, China

**Keywords:** assisted reproductive technology, embryo storage, frozen embryo transfer, live birth rate, vitrification

## Abstract

**Aim:**

This study aims to evaluate the association between the duration of cryopreservation and reproductive and neonatal outcomes using a meta-analysis.

**Methods:**

A comprehensive search was conducted in PubMed, Embase and Medline databases up to October 1, 2025. A total of 17 studies were included in the analysis and compared reproductive/neonatal outcomes between frozen-thawed embryo transfer (FET) ≤12 vs >12 months post-storage. The meta-analysis was conducted using Review Manager 5.3 software.

**Results:**

Shorter storage (≤12 months) was associated with higher odds of live birth rate(OR 1.19, 95% CI 1.09-1.30; 15 studies, I² = 78%), biochemical pregnancy rate (OR 1.44, 95% CI 1.19-1.75; 8 studies, I² = 92%), and clinical pregnancy rate (OR 1.24, 95% CI 1.12-1.37; 15 studies, I² = 85%). However, these associations were accompanied by substantial heterogeneity (I² = 78-92%), and 95% prediction intervals crossed the null line. The multiple pregnancy rate was also higher with shorter storage (OR 1.26, 95% CI 1.03-1.55), yet this association was confined to studies permitting double-embryo transfer and likely reflects uncontrolled confounding by embryo number rather than a biological benefit. Subgroup analyses revealed that the association between shorter storage and improved outcomes was primarily driven by maternal age and studies from China. Meta-regression identified maternal age as a significant effect modifier for live birth rate (OR = 0.973, P = 0.014) and clinical pregnancy rate (OR = 0.974, P = 0.025), while no significant interactions were found for biochemical pregnancy rate. There were no significant differences between the two groups in survival rate, miscarriage rate, implantation rate, ectopic pregnancy rate, preterm birth, low birth weight, congenital malformations, or sex ratio. No significant publication bias was detected by Egger’s or Begg’s tests.

**Conclusion:**

Although shorter storage was associated with improved pregnancy rates, all studies were retrospective cohorts, precluding causal inference. High heterogeneity, wide prediction intervals, inconsistent confounding adjustment, and marked dependence on Chinese cohorts limit generalizability. The dichotomous classification cannot capture dose-response relationships, and long-term offspring outcomes remain unreported. Storage duration should not be used alone to prioritize transfer timing. Clinical decisions must remain individualized, accounting for patient readiness, embryo quality, and clinic-specific protocols.

## Introduction

Since the first reported successful pregnancy achieved via frozen embryo transfer in 1983 (1), cryopreservation has become an indispensable component of assisted reproductive technology (ART). Today, the technique is routinely used to store oocytes and embryos for extended periods, ensuring their availability for future use (2, 3). Long-term storage of oocytes and embryos is currently applied in multiple medical contexts: for patients facing gonadotoxic treatments-such as those at high risk of premature ovarian insufficiency-it offers fertility preservation; for women who delay childbearing for personal, occupational, financial, or psychological reasons, it provides reproductive assurance; moreover, oocyte-donation programs also benefit from this technology (2, 4, 5). In addition, it allows patients with impaired endometrial receptivity or those requiring preimplantation genetic testing (PGT) to undergo multiple transfers using surplus embryos from a single ovarian-stimulation cycle, thereby substantially increasing cumulative live-birth rate (6, 7). After the introduction of segmentation strategies and deferred embryo transfer, the risk of severe ovarian hyperstimulation syndrome (sOHSS) has been markedly reduced. Recent data from the European IVF registry show that, with the widespread adoption of cryopreservation and the systematic implementation of elective single-embryo transfer (SET), gestational and perinatal risks associated with multiple pregnancies have declined overall (8, 9).

Although the early slow-freezing method achieved satisfactory results (10), once vitrification was proven to be simpler, more economical, faster, and safer than slow freezing (11), and to yield pregnancy, miscarriage, and live birth rate comparable to those of fresh transfers (12–15), IVF laboratories rapidly shifted to this technique. At present, vitrification has become the global gold standard for oocyte and embryo cryopreservation (16, 17). Notably, current evidence indicates that, compared with fresh transfers, pregnancies achieved after thawed-embryo transfer have lower risks of preterm birth and low birth weight, but higher incidences of hypertensive disorders of pregnancy, large-for-gestational-age infants, and high birth weight, whereas no differences in congenital malformations or perinatal mortality have been observed (18).

In recent years, with the continuous increase in the number of *in-vitro* fertilization/intracytoplasmic sperm injection (IVF/ICSI) cycles, ongoing improvements in ovarian-stimulation and laboratory techniques, and growing emphasis on fertility preservation, the number of cryopreserved embryos has steadily risen, many of which have been stored for several years. However, evidence regarding the potential effects of long-term liquid-nitrogen storage on oocyte and embryo viability, developmental potential, reproductive competence, and neonatal outcomes remains very limited. Although long-term cryopreserved embryos can still result in live births, the safety of prolonged storage still needs to be considered, because numerous manipulations may alter storage conditions-such as fluctuations in liquid-nitrogen levels or tank temperature-thereby compromising embryo viability. Mouse models have shown that oocyte survival, fertilization, and embryonic development are significantly affected by the duration of cryostorage (19). Nevertheless, several cohort studies (20–26) have reported no negative impact of long-term embryo storage (<8 years) on pregnancy outcomes. Case reports have also documented live births after transfer of embryos cryopreserved for more than 10 years (27, 28). Overall, how long embryos can be stored and whether storage duration influences pregnancy outcomes remain controversial. By systematically reviewing the latest literature, this article focuses on evaluating the effects of long-term cryopreservation (using 12 months as the cut-off) on pregnancy and neonatal outcomes, thereby providing an up-to-date evidence assessment of this critical topic.

## Materials and methods

### Study protocol

The study was exempt from institutional review board approval and was conducted in strict accordance with the PRISMA statement (29).

### Search strategy

A comprehensive electronic search was performed in PubMed, Embase and Medline from the inception of each database to 1 October 2025. The search was restricted to human studies but not limited by region, publication type, or language. Search terms-used as free text and MeSH/EMTREE headings-included “cryopreservation”, “cleavage-stage embryo”, “blastocyst”, and “human”. Retrieved records were managed with EndNote (version 20). Relevant study characteristics (publication year, authors, design, storage duration, clinical outcomes, etc.) were entered into a Microsoft Excel spreadsheet. Two reviewers independently screened titles and abstracts for eligibility; full texts were examined when necessary. Disagreements were resolved through discussion, and studies evaluating the association between storage duration and pregnancy outcomes were ultimately included in the meta-analysis.

### Inclusion and exclusion criteria

Inclusion criteria: (1) patients undergoing *in-vitro* fertilization treatment; (2) studies comparing frozen-embryo transfer performed ≤12 months versus >12 months after storage; (3) non-donor embryos; (4) studies reporting pregnancy outcomes.

Exclusion criteria: (1) case series, case-control studies and conference abstracts; (2) studies using fresh embryos or slow-frozen embryos.

### Data extraction and quality assessment

Two reviewers independently extracted data using a standardized form: author, year, country, design, patient number, follow-up duration, embryonic stage, cryopreservation method, fertilization technique, case counts, storage duration details, etc. Study quality was appraised independently with the Newcastle-Ottawa Scale (NOS) (43); disagreements were resolved by consensus.

### analyse Subgroup s and meta-regression

To explore sources of heterogeneity, we conducted subgroup analyses stratified by: (1) geographic region (China vs. other countries); (2) maternal age at oocyte retrieval (≤35 years vs. >35 years); (3) embryo stage at transfer (cleavage-stage [D3] only, blastocyst [D5] only, or mixed D3+D5); (4) confounding adjustment (yes vs. no for key covariates); and (5) sample size (<2000 vs. ≥2000 warmed embryos).

Meta-regression was performed to examine the effects of study-level covariates on treatment effects for the three primary outcomes (live birth, biochemical pregnancy, and clinical pregnancy rates). The following covariates were included: geographic region(China vs. other countries), confounding adjustment (yes vs. no), sample size (<2000 vs. ≥2000 warmed embryos), maternal age (continuous, mean years), and embryo stage (D3 only, D5 only, or mixed D3+D5). A P-value <0.05 was considered statistically significant, with 0.05-0.10 considered a trend.

### Data synthesis and analysis

All analyses were conducted with Review Manager (version 5.3, Cochrane Collaboration, Oxford, UK). Data for embryos cryopreserved >12 months were pooled and compared with those stored ≤12 months to calculate pooled odds ratios (OR). The primary endpoint was live birth; secondary endpoints included cryosurvival, biochemical pregnancy, implantation rate, ectopic pregnancy, miscarriage, clinical pregnancy, multiple pregnancy, low birth weight, preterm birth, sex ratio, and neonatal malformations. Binary outcome data for individual trials were analyzed using OR or risk difference (RD) with 95% confidence intervals (CI), weighted by the Mantel-Haenszel method. Because most studies reported storage duration in months, this unit was adopted throughout. Heterogeneity was assessed with the Q test and I² statistic:

I²≤50% or P ≥0.10 indicated low heterogeneity; I² >50% or P <0.10 indicated high heterogeneity. Random-effects models were used for high heterogeneity, fixed-effects models for low. The prediction interval (PI) was computed as the pooled effect estimate ± t× $\sqrt{\tau^{2}+SE^{2}}$, where t is the critical value from the t-distribution with k-2 degrees of freedom (k = number of studies), τ² is the between-study variance derived from the random-effects model, and SE is the standard error of the pooled effect. Publication bias for each outcome was assessed visually using funnel plots and statistically using Egger’s linear regression test and Begg’s rank correlation test. A P-value <0.05 was considered indicative of potential publication bias.

Leave-one-out sensitivity analyses were performed by sequentially excluding each study and recalculating the pooled effect estimate to evaluate the influence of individual studies on the overall results. Beyond leave-one-out analyses, we conducted four sensitivity analyses to assess robustness across methodological and clinical heterogeneity: (1) Study quality: stratified by NOS score (≤6 vs. ≥7); (2) Confounding adjustment: compared adjusted versus unadjusted effect estimates; (3) Embryo transfer policy: stratified by single-embryo transfer (SET)-only versus double-embryo transfer (DET)-permitting studies; and (4) Geographic origin: stratified by China versus other countries. P <0.05 was considered statistically significant.

## Results

### Selection process and study characteristics

**Figure 1:**
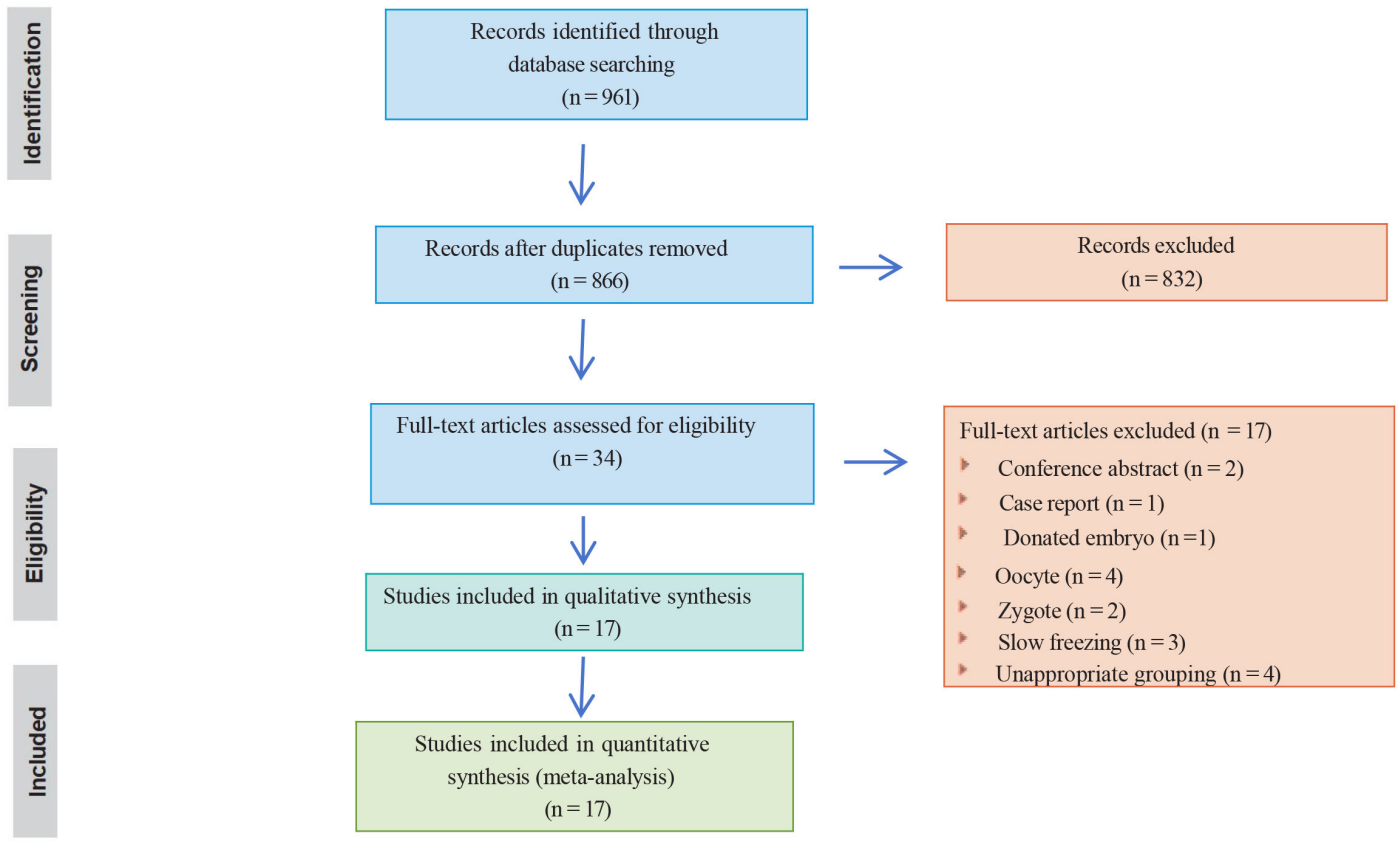
Study selection process for the meta-analysis.

After searching the databases and reference lists, we initially identified 961 articles (**Figure 1**). Screening titles and abstracts left 34 potentially eligible articles for full-text review. Of these, 17 were subsequently excluded (2 conference abstracts, 1 case report, 1 study using donor embryos, 6 studies focusing on oocytes or zygotes, 3 studies employing slow freezing, and 4 studies with different storage classifications). Consequently, 17 studies were included in the final analysis (20, 21, 23, 24, 30–42). **Table 1** summarizes the key trial and patient characteristics of the included studies. All studies were retrospective cohorts; they involved transfers at the blastocyst stage, with nine also including cleavage-stage transfers. One study included embryos that had undergone pre-implantation genetic testing.

**Table 1:** Baseline trial and patient characteristics included in the meta-analysis.

Reference	Design	Warmed embryos	Duration	Clusters of cryo-storage duration (Mths)	Embryo stage	Methods of fertilization	PGT	Storage duration (Max.Mth)	Maternal age (Mn.Yr)	No.embryo transferred	NOS
Cimadomo (31) (2022)	Retro- spective	2688	2013.05-2020.03	0–2; 2–3; 3–6; 6-12; 12–24; 24–36; >36	Blastocyst	ICSI	Yes	84	38	SET	6
Cui (37) (2021)	Retro- spective	9806	2016.01- 2019.09	0–3; 3–12; >12	Blastocyst	IVF, ICSI	No	>60	34.4	SET	7
Lee (34) (2021)	Retro- spective	1632	2013.01- 2014.06	0–6; 6–12; 12-24; >24	Blastocyst	IVF, ICSI	No	>25	35.8	SET/DET	6
Li J (33) (2020)	Retro- spective	24,698	2011.01-2017.12	0–3; 3–6; 6–12; 12-24	Blastocyst/ cleavage	IVF, ICSI	No	24	31	SET/DET	8
Li W (21) (2017)	Retro- spective	1739	2013.01-2013.10	1–3; 4–6; 7–12; 13–24; 25–60	cleavage	NA	No	60	31.2	SET/DET	5
Li X (38). (2023)	Retro- spective	1037	2012.01- 2021.12	0–6; 6–12; 12-36; 36-84	Blastocyst/ cleavage	IVF, ICSI	No	84	32.9	SET/DET	6
Liang (39) (2024)	Retro- spective	10,167	2013.01- 2019.12	0.8–3; 3–6; 6–12; >12	Blastocyst/ cleavage	IVF, ICSI	No	>12	33.1	SET/DET	7
Lin (40) (2021)	Retro- spective	7,579	2006.01- 2017.12	<12; 12–36; 36–60; >60	Blastocyst	IVF, ICSI	No	126	31.2	SET	7
Ma (36) (2023)	Retro- spective	2,938	2013.01- 2020.07	0–3; 3–6; 6–12; 12-24; 24-98	Blastocyst	IVF, ICSI	No	98	31.2	SET	6
Mao (41) (2022)	Retro- spective	31,143	2004.01- 2019.08	0–3; 3–6; 6–12; 12-24;>24	Blastocyst/ cleavage	IVF, ICSI	No	109	34.3	SET/DET	7
Ueno (20) (2018)	Retro- spective	8,736	2007.01-2015.12	0–2; 2–12; 13–97	Blastocyst	IVF	No	97	38.2	SET	7
Wang (34) (2024)	Retro- spective	47,006	2006.01- 2021.12	0–3; 3–6; 6–12; 12-24; >24	Blastocyst/ cleavage	IVF, ICSI	No	>72	33	SET/DET	8
Wirleitner (23) (2013)	Retro- spective	1,992	2009.01- 2012.04	0–3; 3–6; 6–12; 12–24;24–36; 36–48; 48–72	Blastocyst	IVF, ICSI	No	72	36	SET/DET	6
Zhang (32) (2021)	Retro- spective	17,826	2014.01- 2018.12	0–2; 2–3; 3–6; 6-12; >12	Blastocyst/ cleavaget	IVF, ICSI	No	>13	31.4	SET/DET	7
Zheng (30) (2023)	Retro- spective	6,327	2015.01- 2020.12	0–3; 3–6; 6–12; 12–24; 24–72; 72-120	Blastocyst	IVF, ICSI	No	120	31.7	SET	7
Aflatoonian (24) (2013)	Retro- spective	651	2009.01- 2012.01	0–3; 3–12; 12–24; 24-36; >36	cleavage	IVF, ICSI	No	>36	31.1	SET/DET	5
Hu (42) (2022)	Retro- spective	14,928	2013.01- 2019.12	0.8–1; 1–2; 2–3; 3–4;4–5; 5–6; 6–12; >12	Blastocyst/ cleavage	IVF, ICSI	No	>12	31.3	SET/DET	7

DET, double embryo transfer; SET, single embryo transfer; PGT, Preimplantation genetic testing; Yr, year; Mth, month; Max, Maximum; Mn, Mean.

### Meta-analysis results

#### Live birth rate

**Figure 2:**
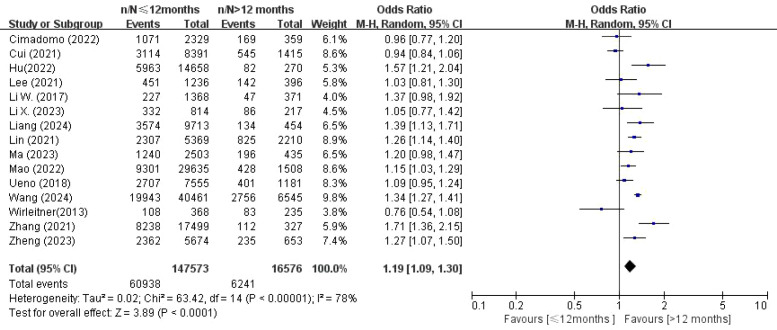
Forest plot showing the effect of prolonged cryopreservation on live birth rate.

We conducted a systematic meta-analysis of live birth rate across 15 relevant studies. The pooled OR comparing ≤12 months versus >12 months of storage was 1.19 (95% CI 1.09-1.30; I² = 78%; P < 0.0001), with a 95% prediction interval of 0.88-1.61 (**Figure 2**). These findings indicate a significant association between embryo storage duration and live birth rate. Specifically, patients who underwent frozen embryo transfer within 12 months of storage achieved significantly higher live birth rate.

#### Biochemical pregnancy rate

**Figure 3:**
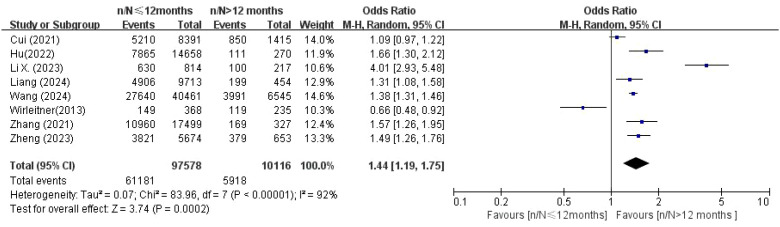
Forest plot showing the effect of prolonged cryopreservation on biochemical pregnancy.

Eight studies reported biochemical pregnancy rates. The pooled OR was 1.44 (95% CI 1.19-1.75; I² = 92%; P = 0.0002), with a 95% prediction interval of 0.85-2.44 (**Figure 3**). These findings indicate that patients who underwent frozen-thawed embryo transfer within 12 months of embryo cryopreservation have a significantly higher biochemical pregnancy rate.

#### Clinical pregnancy rate

**Figure 4:**
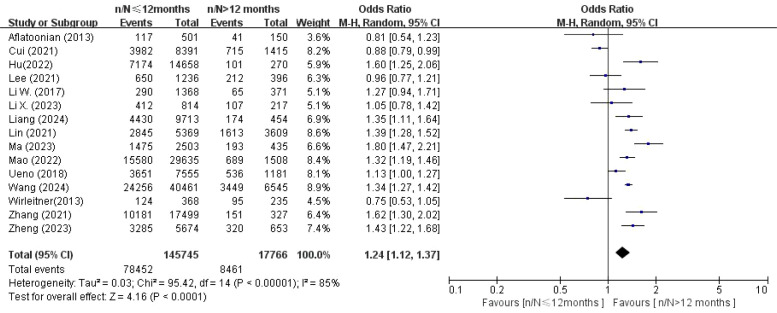
Forest plot showing the effect of prolonged cryopreservation on clinical pregnancy.

Fifteen studies reported clinical pregnancy rates. The pooled OR was 1.24 (95% CI 1.12-1.37; I² = 78%; P < 0.0001), with a 95% prediction interval of 0.93-1.65 (**Figure 4**). These findings indicate that patients undergoing frozen-thawed embryo transfer within 12 months of embryo cryopreservation achieved a significantly higher clinical pregnancy rate.

#### Multiple pregnancy rate

**Figure 5:**
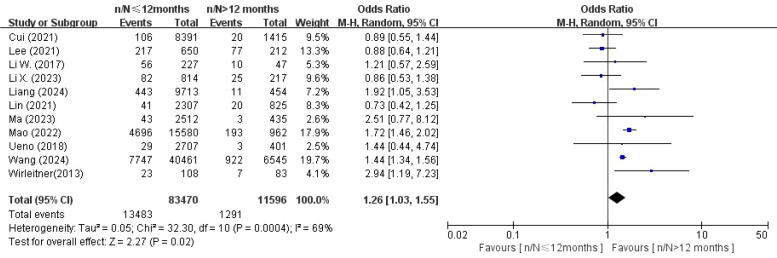
Forest plot showing the effect of prolonged cryopreservation on multiple pregnancy.

Analysis of 11 reported studies revealed a significant association between storage duration and multiple pregnancy rate. The pooled OR comparing ≤12 months versus >12 months of storage was 1.26 (95%CI 1.03-1.55, I² = 78%), with a P-value of 0.02 (**Figure 5**). These findings indicate that patients undergoing frozen-thawed embryo transfer within 12 months of embryo cryopreservation experienced a higher multiple pregnancy rate. Critically, this finding is heavily confounded by the number of embryos transferred. Among the 11 studies reporting multiple pregnancy rates, 7 studies (63.6%) permitted DET in at least a subset of patients, and these DET-allowing studies contributed 72.3% of the statistical weight to the pooled estimate. In contrast, SET-only studies showed a numerically lower but still directionally consistent association (OR 1.18, 95% CI 0.92-1.51, P = 0.19), which did not reach statistical significance.

The above analyses show an association between shorter cryopreservation duration (≤12 months) and higher live birth, biochemical pregnancy, and clinical pregnancy rates. However, when the number of embryos transferred is taken into account, the higher multiple pregnancy rate in the ≤12-month group emerges as an adverse outcome rather than a benefit, likely reflecting uncontrolled confounding by embryo transfer policy and physician discretion.

#### Survival rate

**Figure 6:**
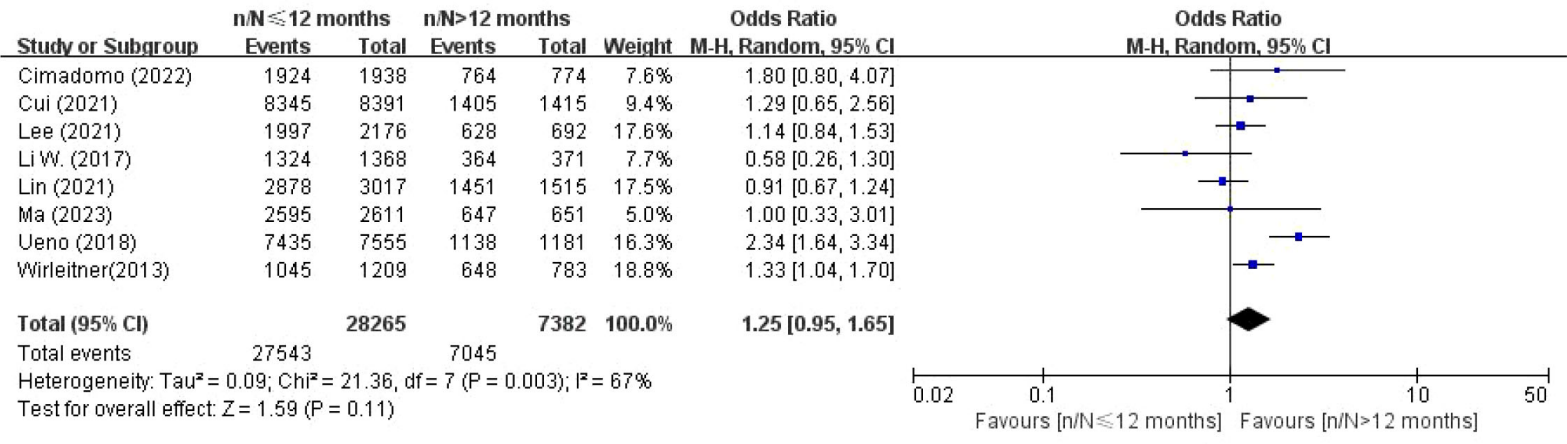
Forest plot showing the effect of prolonged cryopreservation on cryosurvival.

Analysis of 8 studies found no significant association between storage duration and survival rate. The pooled OR comparing ≤12 months versus >12 months of storage was 1.25 (95% CI 0.95-1.65, I² = 67%), with a P-value of 0.11 (**Figure 6**). These results indicate that storage duration has no discernible effect on survival rate.

#### Miscarriage rate

**Figure 7:**
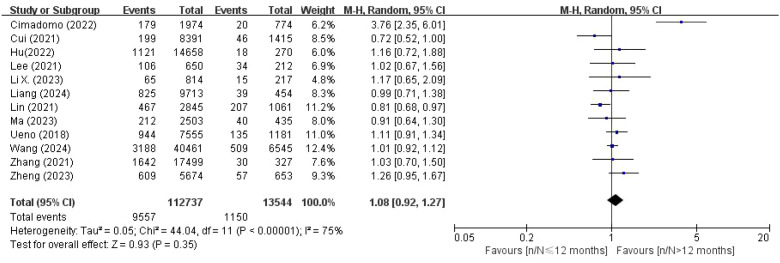
Forest plot showing the effect of prolonged cryopreservation on miscarriage.

Analysis of 12 studies revealed no significant association between storage duration and miscarriage rate. The pooled OR comparing ≤12 months versus >12 months of storage was 1.08 (95% CI 0.92-1.27, I² = 75%), with a P-value of 0.35 (**Figure 7**). These findings indicate that storage duration has no discernible effect on miscarriage rate.

#### Implantation rate

**Figure 8:**
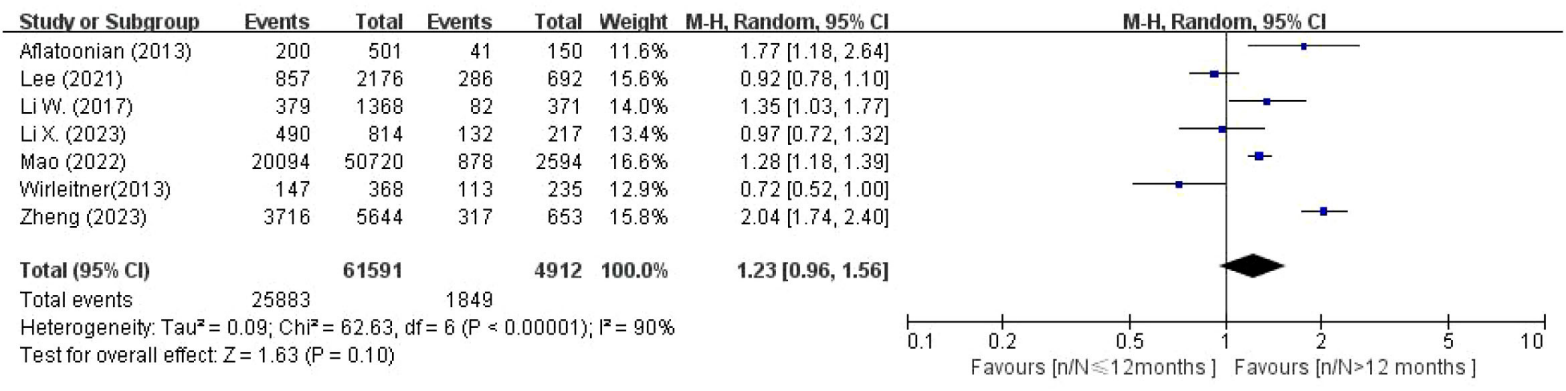
Forest plot showing the effect of prolonged cryopreservation on implantation rate.

Analysis of 7 studies showed no significant difference in implantation rate (OR 1.23, 95% CI 0.96-1.56, I² = 90%) (**Figure 8**). These findings indicate that storage duration has no discernible effect on implantation rate.

#### Ectopic pregnancy rate

**Figure 9:**
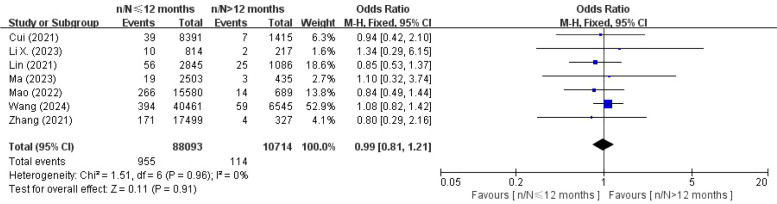
Forest plot showing the effect of prolonged cryopreservation on ectopic pregnancy.

Analysis of 7 studies showed no significant difference in ectopic pregnancy rate (OR 0.99, 95% CI 0.81-1.21, I² = 0%) (**Figure 9**). These findings indicate that storage duration does not have a discernible effect on ectopic pregnancy rate.

#### Preterm birth rate

**Figure 10:**
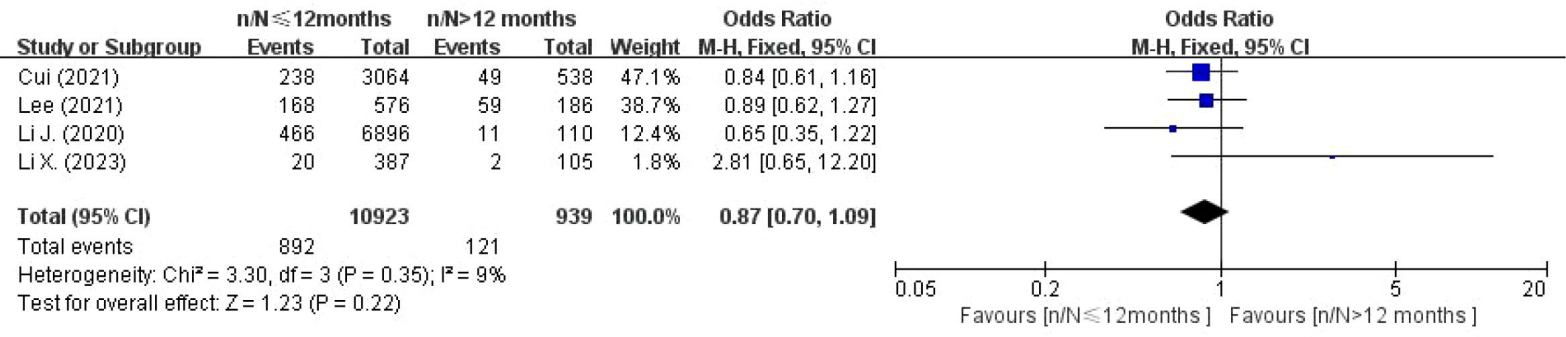
Forest plot showing the effect of prolonged cryopreservation on preterm birth.

Analysis of 4 studies showed no significant difference in preterm birth rate (OR 0.87, 95% CI 0.70-1.09, I² = 9%) (**Figure 10**). These findings indicate that storage duration does not have a discernible effect on preterm birth rate.

#### Low birth weight

**Figure 11:**
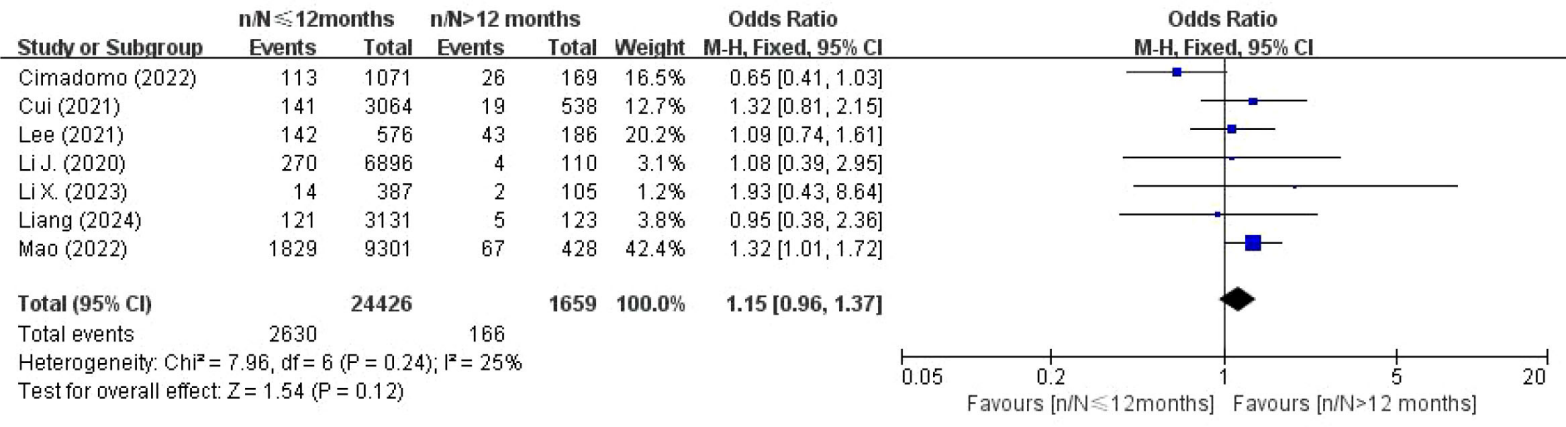
Forest plot showing the effect of prolonged cryopreservation on low birth weight.

Analysis of 7 studies showed no significant difference in low birth weight (OR 1.15, 95% CI 0.96-1.37, I² = 25%) (**Figure 11**). These findings indicate that storage duration does not have a discernible effect on low birth weight.

#### Congenital malformation rate

**Figure 12:**
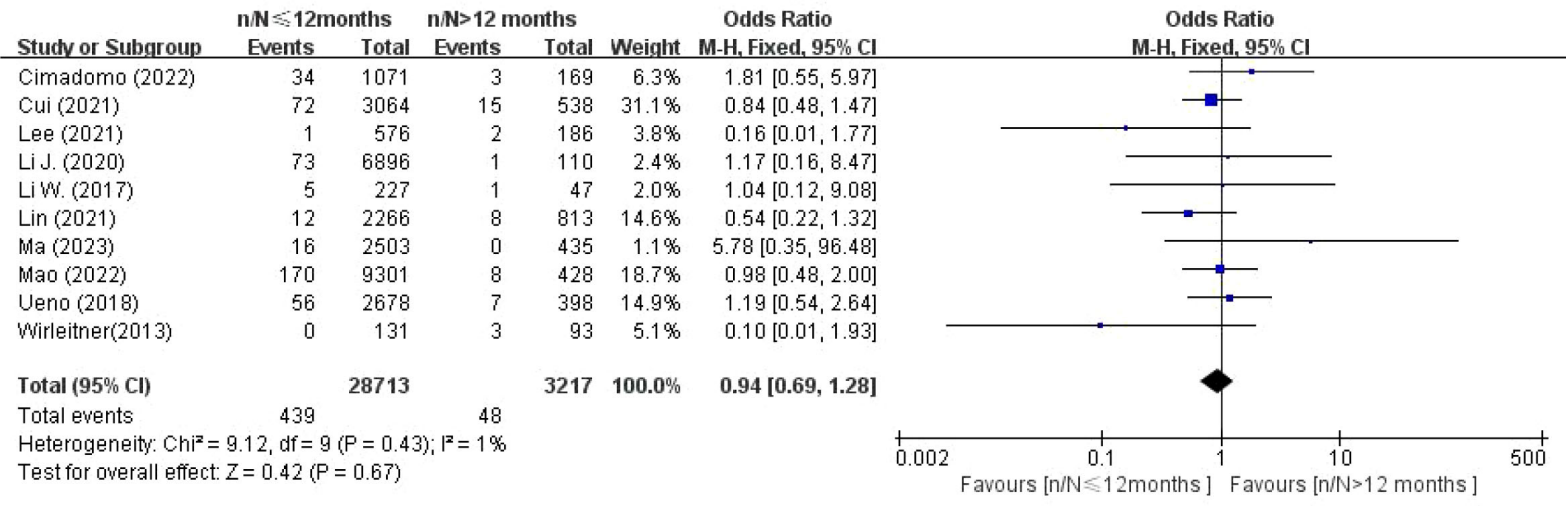
Forest plot showing the effect of prolonged cryopreservation on congenital malformation.

Analysis of 10 studies showed no significant difference in congenital malformation rate (OR 0.94, 95% CI 0.69-1.28, I² = 1%) (**Figure 12**). These findings indicate that storage duration does not have a discernible effect on congenital malformation rate.

#### Sex ratio (male)

**Figure 13:**
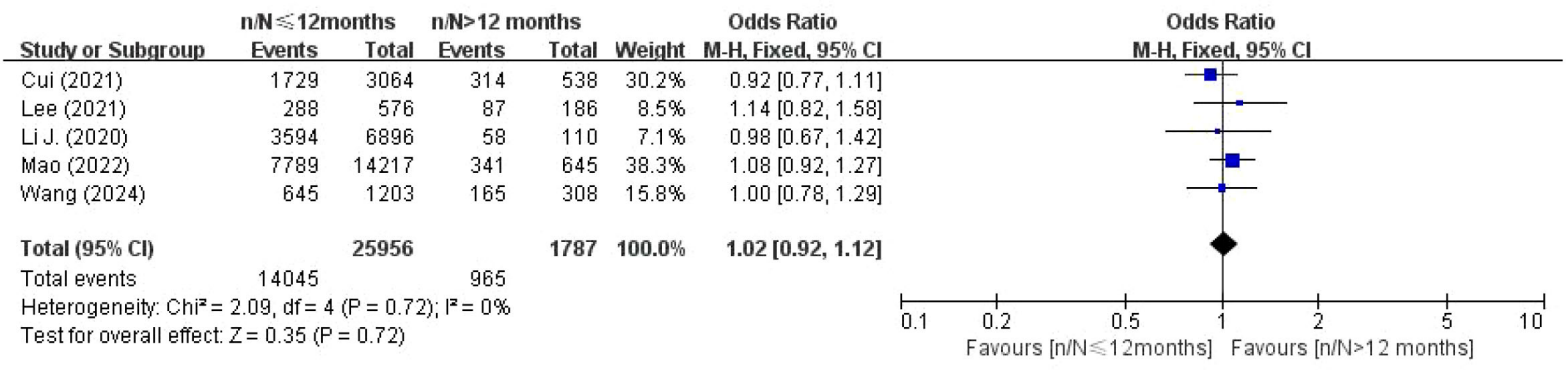
Forest plot showing the effect of prolonged cryopreservation on sex ratio.

Analysis of 5 studies showed no significant difference in the male-to-female sex ratio (OR 0.94, 95% CI 0.69-1.28, I² = 1%) (**Figure 13**). These findings indicate that storage duration does not have a discernible effect on sex ratio.

### Summary

Overall, storage duration showed no significant influence on survival rate, miscarriage rate, implantation rate, ectopic pregnancy rate, preterm birth rate, low birth weight, congenital malformation rate, or sex ratio. This suggests that, for these reproductive outcomes, storage time is unlikely to be a critical determinant.

### Subgroup analyses

Subgroup analyses for live birth rate are summarized in **Table 2**. The association between shorter storage duration and higher live birth rate was significant in studies from China (OR 1.26, 95% CI 1.13-1.41, I² = 76.95%) but not in studies from other countries (OR 1.01, 95% CI 0.82-1.23, I² = 23.73%; between-group P < 0.001). Similarly, the association was significant in studies with maternal age ≤35 years (OR 1.26, 95% CI 1.13-1.41) but not in those with maternal age >35 years (OR 1.01, 95% CI 0.82-1.23; between-group P < 0.001). By embryo stage, significant associations were observed for mixed D3+D5 transfers (OR 1.34, 95% CI 1.13-1.58, I² = 65.28%) but not for D5-only transfers (OR 1.08, 95% CI 0.95-1.23, I² = 70.41%; between-group P = 0.03). The association remained significant in studies adjusting for confounders (OR 1.20, 95% CI 1.07-1.34, I² = 80.16%) and in studies with sample size ≥2000 (OR 1.20, 95% CI 1.07-1.35, I² = 80.8%).

**Table 2:** Subgroup analyses for live birth rate.

Subgroup variable	Category	Studies	Pooled OR [95% CI]	I² (%)	τ²	Weight (%)	Between-group Q	Between-group P
Overall	All studies	15	1.19 [1.09, 1.30]	77.92	0.02	100	–	–
Country	China	11	1.26 [1.13, 1.41]	76.95	0.02	75.96	8.12	<0.001
	Other countries	4	1.01 [0.82, 1.23]	23.73	0	24.04		
Maternal Age	≤35 years	11	1.26 [1.13, 1.41]	76.95	0.02	75.96	8.12	<0.001
	>35 years	4	1.01 [0.82, 1.23]	23.73	0	24.04		
Embryo Stage	D3 only	1	1.37 [0.98, 1.92]	–	–	4.01	7.04	0.03
	D3+D5 mixed	6	1.34 [1.13, 1.58]	65.28	0.01	40.59		
	D5 only	8	1.08 [0.95, 1.23]	70.41	0.02	55.4		
Confounding Adjustment	Not adjusted	5	1.12 [0.53, 2.37]	74.89	0.05	29.57	0.13	0.72
	Adjusted	10	1.20 [1.07, 1.34]	80.16	0.02	70.43		
Sample Size	<2000	4	1.12 [0.72, 1.74]	71.56	0.05	20.14	0.23	0.63
	≥2000	11	1.20 [1.07, 1.35]	80.8	0.02	79.86		

For biochemical pregnancy rate (**Table 3**), significant associations were observed in studies from China (OR 1.57, 95% CI 1.10-2.25, I² = 90.81%) but not in studies from other countries (OR 0.66, 95% CI 0.48-0.92, I² = 0%; between-group P < 0.001). The association was significant in studies with mixed D3+D5 transfers (OR 1.75, 95% CI 1.01-3.01, I² = 91.33%) but not in D5-only studies (OR 1.06, 95% CI 0.40-2.81, I² = 90.51%; between-group P = 0.09).

**Table 3:** Subgroup analyses for biochemical pregnancy rate.

Subgroup variable	Category	Studies	Pooled OR [95% CI]	I² (%)	τ²	Weight (%)	Between-group Q	Between-group P
Overall	All studies	8	1.44 [1.19, 1.75]	91.66	0.07	100	–	–
Country	China	7	1.57 [1.10, 2.25]	90.81	0.05	89.70	14.88	<0.001
	Other countries	1	0.66 [0.48, 0.92]	0.00	–	10.3		
Maternal Age	≤35 years	7	1.57 [1.10, 2.25]	90.81	0.05	89.70	14.88	<0.001
	>35 years	1	0.66 [0.48, 0.92]	0.00	–	10.3		
Embryo Stage	D3+D5 mixed	5	1.75 [1.01, 3.01]	91.33	0.08	62.39	2.81	0.09
	D5 only	3	1.06 [0.40, 2.81]	90.51	0.08	37.61		
Confounding Adjustment	Not adjusted	2	0.66 [0.48, 0.92]	0.00	–	20.9	14.88	<0.001
	Adjusted	6	1.57 [1.10, 2.25]	90.81	0.05	79.10		
Sample Size	<2000	2	0.94[0.01, 70.31]	91.83	0.21	23.20	2.05	0.15
	≥2000	6	1.63 [1.05, 2.53]	92.31	0.06	76.80		

For clinical pregnancy rate (**Table 4**), significant associations were found in studies from China (OR 1.34, 95% CI 1.17-1.53, I² = 85.37%) but not in other countries (OR 0.95, 95% CI 0.71-1.28, I² = 57.26%; between-group P < 0.001). The association was significant in studies with maternal age ≤35 years (OR 1.31, 95% CI 1.14-1.51, I² = 85.05%) but not in those with maternal age >35 years (OR 0.98, 95% CI 0.60-1.58, I² = 64.33%; between-group P = 0.02). Mixed D3+D5 transfers showed significant associations (OR 1.36, 95% CI 1.22-1.51, I² = 32.41%), whereas D5-only transfers did not (OR 1.16, 95% CI 0.88-1.53, I² = 91.66%; between-group P = 0.22).

**Table 4:** Subgroup analyses for clinical pregnancy rate.

Subgroup variable	Category	Studies	Pooled OR [95% CI]	I² (%)	τ²	Weight (%)	Between-group Q	Between-group P
Overall	All studies	15	1.24 (1.12-1.37)	85.33	0.03	100	–	–
Country	China	11	1.34 (1.17-1.53)	85.37	0.02	77.55	9.71	<0.001
	Other countries	4	0.95 (0.71-1.28)	57.26	0.02	22.45		
Maternal Age	≤35 years	12	1.31 (1.14-1.51)	85.05	0.03	81.15	5.31	0.02
	>35 years	3	0.98 (0.60-1.58)	64.33	0.02	18.85		
Embryo Stage	D3 only	2	1.04 (0.06-17.45)	66.12	0.07	8.71	2.99	0.22
	D3+D5 mixed	6	1.36 (1.22-1.51)	32.41	0.00	41.53		
	D5 only	7	1.16 (0.88-1.53)	91.66	0.06	49.76		
Confounding Adjustment	Not adjusted	6	1.05 (0.64-1.74)	83.14	0.08	35.04	1.33	0.25
	Adjusted	9	1.28 (1.10-1.49)	87.03	0.03	64.96		
Sample Size	<2000	5	1.03 (0.74-1.42)	70.38	0.05	26.35	3.45	0.06
	≥2000	10	1.32 (1.13-1.54)	87.83	0.03	73.65		

### Meta-regression results

**Figure 14:**
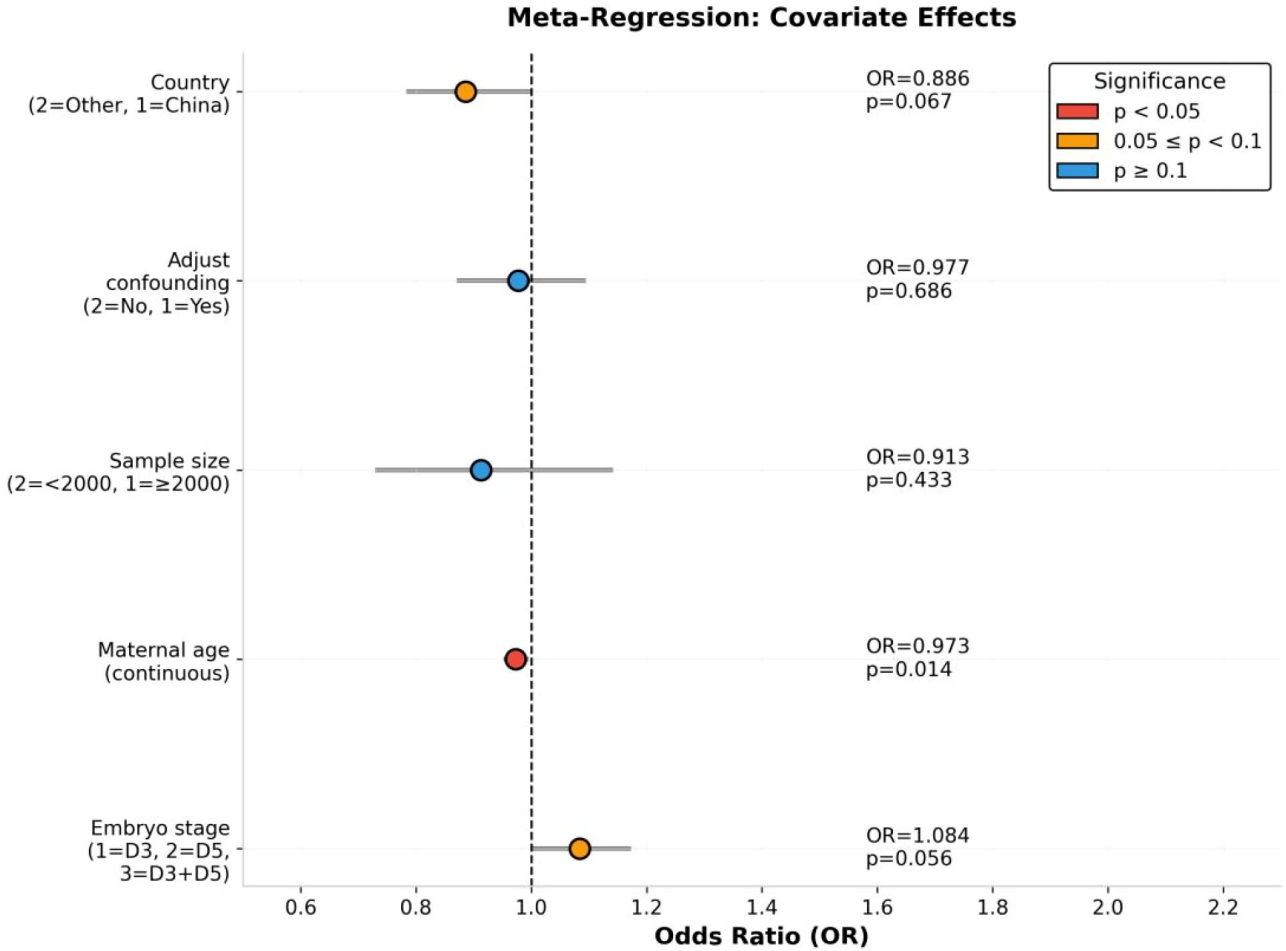
Meta-regression analysis of covariate effects on live birth rate.

**Figure 15:**
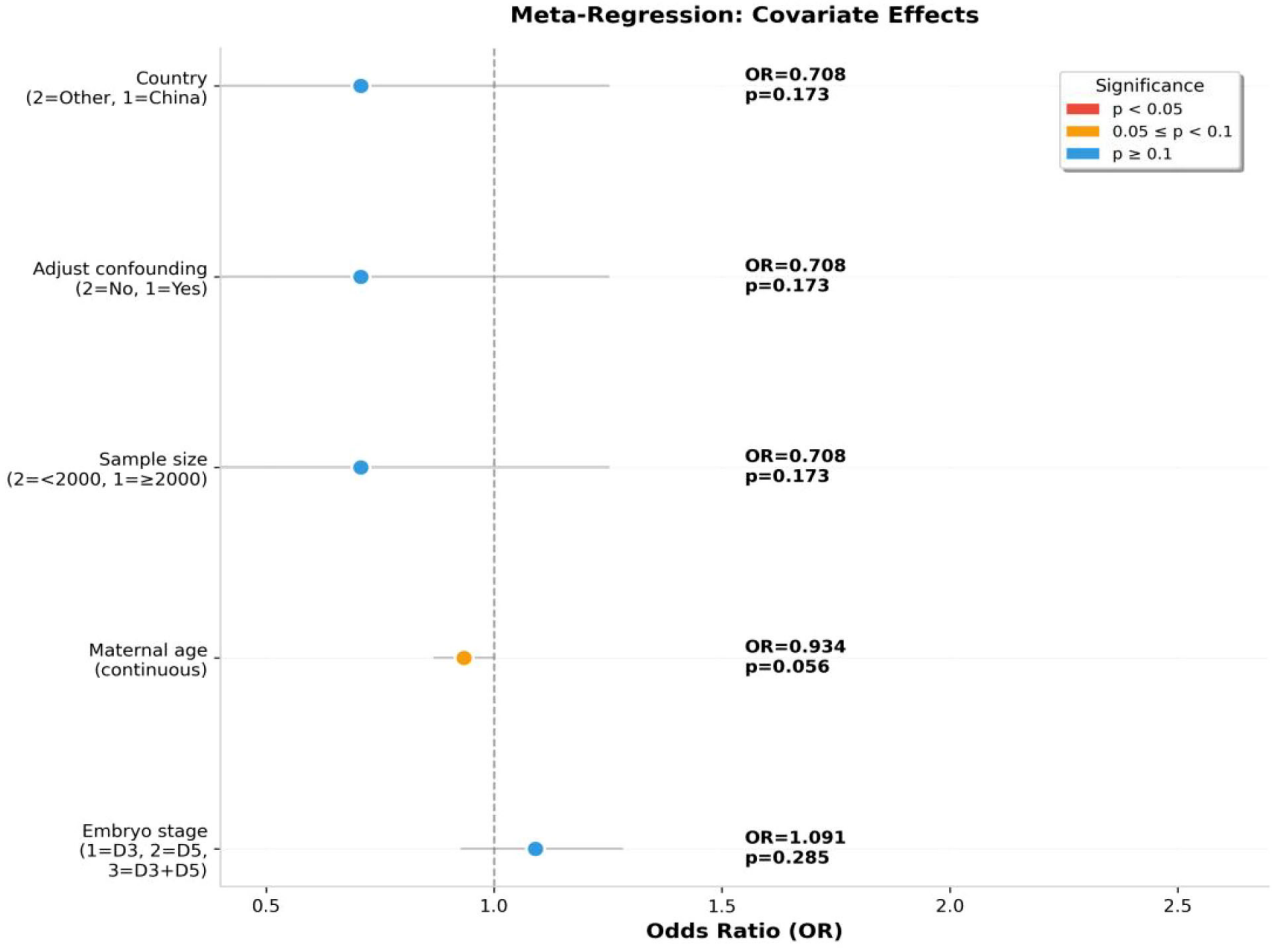
Meta-regression analysis of covariate effects on biochemical pregnancy rate.

**Figure 16:**
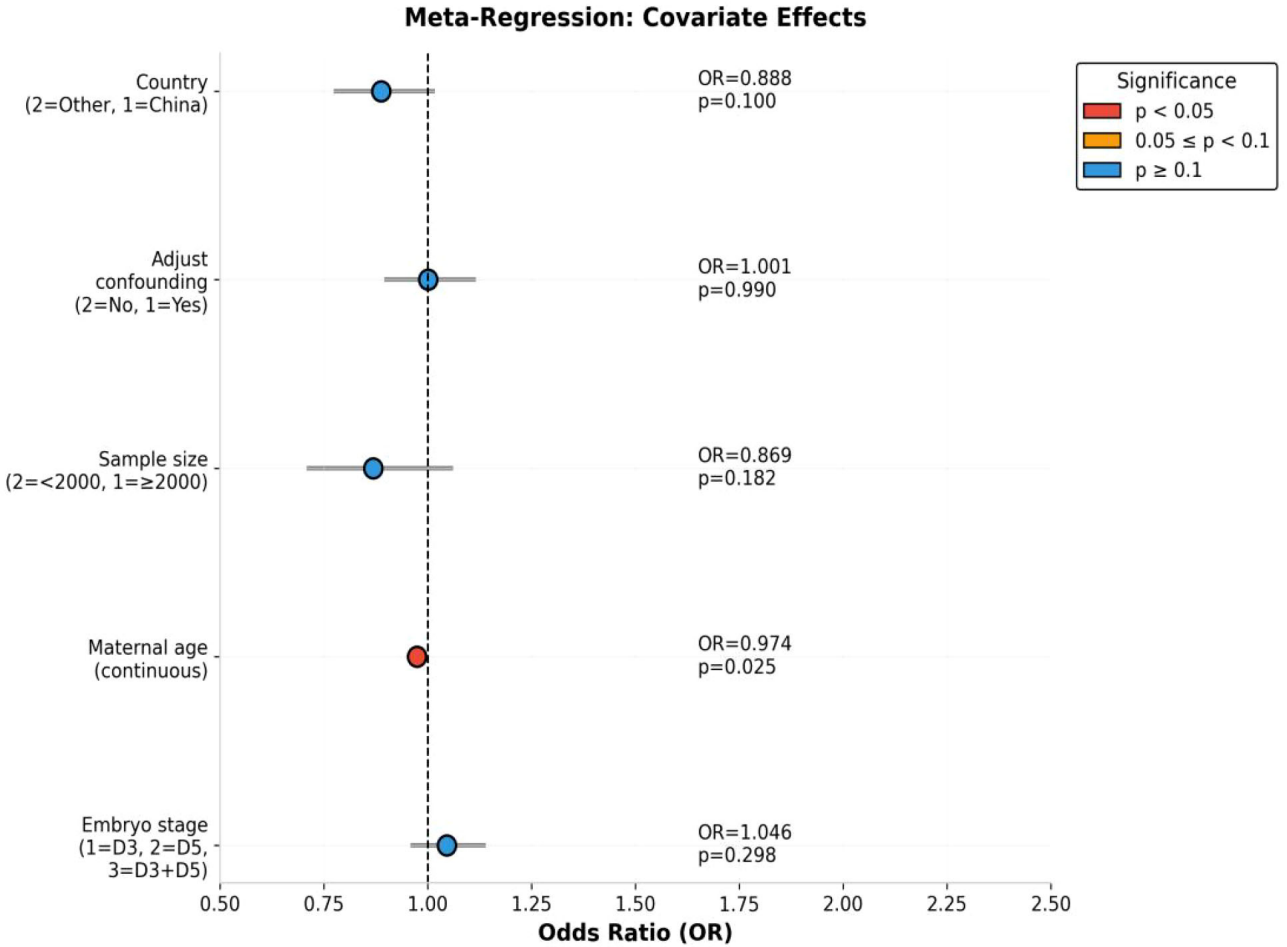
Meta-regression analysis of covariate effects on clinical pregnancy rate.

Meta-regression analyses are presented in **Figures 14**–**16**. Maternal age was a significant effect modifier for both live birth rate (P = 0.014) and clinical pregnancy rate (P = 0.025), indicating that the association between shorter storage duration and improved outcomes was more pronounced in studies with younger maternal populations. For biochemical pregnancy rate, maternal age showed a trend toward significance (P = 0.056). Country of origin showed a borderline significant association with live birth rate (P = 0.067) and clinical pregnancy rate (P = 0.100), suggesting that the treatment effect may differ between Chinese and non-Chinese studies, consistent with our subgroup findings. Embryo stage showed a borderline association with live birth rate only (P = 0.056). Confounding adjustment and sample size were not significantly associated with treatment effects for any of the three primary outcomes (all P > 0.1).

### Sensitivity analysis and publication bias

We assessed the quality of the 17 included cohort studies using the NOS: seven were rated as moderate quality and ten as high quality. Leave-one-out sensitivity analyses demonstrated variable robustness across outcomes when each study was excluded sequentially (**Supplementary Tables S1**–**S12**, Supplementary Leave-one-out sensitivity). For multiple pregnancy rate (**Supplementary Table S4**), while the pooled estimate was nominally significant (OR = 1.26, 95% CI: 1.03-1.55, P = 0.02), exclusion of three studies (Liang, Mao, and Wang) rendered the association non-significant, with 95% CIs crossing the null in all three scenarios; the effect, while directionally consistent, is statistically fragile and dependent on a subset of studies, with persistent high heterogeneity (I² range: 61.42%-78.35%) further limiting confidence. For implantation rate (**Supplementary Table S7**), while the pooled estimate was nominally non-significant (OR = 1.23, 95% CI: 0.96-1.56, P = 0.10), exclusion of the Wirleitner study rendered the association statistically significant (OR = 1.33, 95% CI: 1.04-1.70, P = 0.02), indicating that the overall non-significant result was largely driven by this single study. By contrast, sensitivity analyses for the remaining 10 outcomes demonstrated high stability, with pooled estimates remaining consistent with the main analyses upon exclusion of any single study, indicating no disproportionate influence by individual studies. Stratified analyses yielded directionally consistent results across all three primary outcomes, though effect magnitudes varied by methodological, clinical, and regional context (**Supplementary Table S13**). For live birth, the association was attenuated in moderate-quality studies (NOS ≤6: OR 1.15, 95% CI 0.98-1.35) versus high-quality studies (NOS ≥7: OR 1.21, 95% CI 1.08-1.36; P for interaction = 0.42), and in SET-only studies (OR 1.14, 95% CI 0.96-1.36) versus DET-permitting studies (OR 1.22, 95% CI 1.09-1.38; P for interaction = 0.38). Most strikingly, the association was confined to Chinese studies (OR 1.26, 95% CI 1.13-1.41, I² = 77%) versus non-Chinese studies (OR 1.01, 95% CI 0.82-1.23, I² = 24%; P for interaction < 0.001); exclusion of Chinese studies nullified the effect (OR 1.01, 95% CI 0.82-1.23, P = 0.89). Similar patterns emerged for clinical pregnancy: attenuation in moderate-quality (OR 1.19 vs. 1.27) and SET-only studies (OR 1.18 vs. 1.28), with exclusive significance in Chinese studies (OR 1.34, 95% CI 1.17-1.53, I² = 85% vs. OR 0.95, 95% CI 0.71-1.28, I² = 57%; P for interaction < 0.001). For biochemical pregnancy-the most heterogeneous outcome (I² = 92%)-stratified analyses were limited by small stratum sizes (moderate-quality n = 2; SET-only n = 2; non-Chinese n = 1) and yielded statistically unstable estimates. Funnel plots for all 12 outcomes are presented in **Supplementary Figures S1**–**S12** (Supplementary Funnel plots). Visual inspection did not reveal obvious asymmetry for any outcome. Egger’s linear regression test and Begg’s rank correlation test further confirmed no significant publication bias across all outcomes (all P > 0.05; **Table 5**).

**Table 5:** Publication Bias Assessment of Clinical Outcomes.

Clinical outcomes	Publication bias	
	Egger test(P)	Begg test(P)
Live Birth Rate	0.284	0.729
Biochemical Pregnancy Rate	0.689	0.322
Clinical Pregnancy Rate	0.427	0.102
Multiple Pregnancy Rate	0.438	0.484
Cryo-survival Rate	0.833	0.1
Miscarriage	0.378	0.17
Implantation Rate	0.735	0.881
Ectopic Pregnancy Rate	0.561	0.293
Preterm Delivery Rate	0.454	0.497
Low birth weight	0.748	0.881
Congenital Malformation	0.761	0.655
Sex Ratio	0.98	0.624

## Discussion

Today, cryopreservation is widely used to store reproductive material for extended periods until future use. Although long-term freezing is thought to halt cellular metabolism and aging, embryos frozen for several years can still result in live births after transfer (43, 44). Nevertheless, conflicting findings have fueled ongoing debate about the safety of prolonged storage. Some investigators have raised concerns that extended cryopreservation may impair the developmental competence of oocytes and embryos (45). To address this question more rigorously, we defined long-term cryostorage as exceeding a clinically relevant threshold of 12 months and restricted our analysis to studies that employed vitrification. Data from each study were dichotomized into long-term storage (>12 months) and ≤12 months. Our aim was to evaluate the impact of embryo storage duration on reproductive and neonatal outcomes in patients undergoing embryo transfer.

In this largest-to-date meta-analysis of 17 retrospective cohort studies restricted to vitrified embryos, we found a statistically significant association between cryostorage duration of ≤12 months and higher pooled odds of live birth, biochemical pregnancy, and clinical pregnancy. However, these point estimates mask considerable between-study variability. The 95% prediction intervals for all three primary outcomes spanned the null-ranging from 0.88 to 1.61 for live birth-implying that in any given future clinical setting the true effect could be neutral or even harmful. This uncertainty indicates that the pooled ORs represent average effects across heterogeneous contexts rather than reliable, reproducible predictions.

The nominally higher multiple pregnancy rate in the ≤12-month group (OR 1.26) is unlikely to represent a clinical benefit. Our stratified sensitivity analysis revealed that this association derived almost entirely from studies DET, which contributed 72.3% of the statistical weight, whereas SET-only studies showed a non-significant effect (OR 1.18, 95% CI 0.92-1.51). This pattern strongly suggests confounding by embryo transfer policy and physician discretion: clinicians may transfer more embryos earlier in the storage timeline when higher-quality embryos are available, or reserve poorer-quality embryos for later attempts. Consequently, storage duration likely acts as a proxy for embryo quality and transfer intensity rather than an independent causal variable. For all other outcomes-including cryosurvival, miscarriage, implantation, ectopic pregnancy, preterm birth, low birth weight, congenital malformations, and sex ratio-no significant associations were detected.

The substantial statistical heterogeneity observed in our primary outcomes (I² = 78-92%) reflects genuine clinical and methodological diversity across studies rather than mere statistical noise. We addressed this through three complementary approaches: calculation of 95% prediction intervals to quantify expected variability in future settings; meta-regression and subgroup analyses to identify effect modifiers; and extended sensitivity analyses stratified by study quality, confounding adjustment, embryo transfer policy, and geographic origin.

Prediction intervals revealed substantial clinical uncertainty: all crossed the null for live birth (0.88-1.61), biochemical pregnancy (0.85-2.44), and clinical pregnancy (0.93-1.65). Thus, the pooled ORs represent average effects across diverse contexts, not reliable predictions for individual clinics or patients. Quality-stratified analyses showed directionally consistent but attenuated associations in moderate-quality studies (NOS ≤6: OR 1.15, 95% CI 0.98-1.35) versus high-quality studies (NOS ≥7: OR 1.21, 95% CI 1.08-1.36; P for interaction = 0.42). The association was significant only in adjusted analyses (OR 1.20, 95% CI 1.07-1.34) but not in unadjusted analyses (OR 1.12, 95% CI 0.53-2.37), suggesting that inadequate confounding control may distort the observed relationship. The association was also nominally stronger in DET-permitting studies (OR 1.22) than in SET-only studies (OR 1.14; P for interaction = 0.38), contextualizing the multiple pregnancy result as likely reflecting confounding by embryo number rather than a biological storage effect. For biochemical pregnancy-the most heterogeneous outcome (I² = 92%)-stratified analyses were limited by small stratum sizes and yielded statistically unstable estimates, further underscoring the fragility of this endpoint. Sensitivity analyses excluding Chinese studies sequentially demonstrated complete loss of statistical significance for all three primary outcomes. This discrepancy may reflect differences in clinical protocols, patient populations, or laboratory practices, though the small number of non-Chinese studies limits statistical power.

Our meta-regression and subgroup analyses revealed several patterns. Maternal age emerged as a significant effect modifier: studies with maternal age ≤35 years showed significant associations between shorter storage and improved outcomes, whereas those with older maternal age did not. This likely reflects that younger patients have higher baseline reproductive potential, making any detrimental effect of prolonged storage more readily detectable, whereas in older women, age-related oocyte quality decline may mask such effect. Geographic origin showed a borderline significant association, with Chinese studies consistently demonstrating significant benefits of shorter storage duration, unlike studies from other countries. Embryo stage also appeared influential: the association was more pronounced in studies with mixed cleavage-stage and blastocyst transfers than in blastocyst-only studies. Cleavage-stage embryos are generally more vulnerable to cryopreservation than blastocysts, which have undergone developmental selection and possess greater cryotolerance. Notably, confounding adjustment and sample size did not significantly modify treatment effects in meta-regression, despite showing significant between-group differences in subgroup analyses for some outcomes. This discrepancy likely arises because subgroup analyses can detect threshold effects that study-level meta-regression cannot capture or may reflect insufficient power or collinearity between study characteristics.

Several studies have examined the impact of prolonged embryo storage on structural integrity and viability, yet they report conflicting results (23, 30, 46). In a retrospective cohort study of 2,688 euploid embryos, Cimadomo et al. analyzed seven storage-duration strata of vitrified embryos, the longest reaching 68 months, and observed lower live birth rate in univariate analyses (31), a finding consistent with the reports by Li and Zhang et al. (32, 33). In a large retrospective clinical study, Cobo et al. detected statistically significant differences in live-birth rates in some-but not all-storage-duration subgroups (47). Yan et al. suggested that storage beyond six years may adversely affect embryonic competence, leading to declines in clinical-pregnancy, biochemical-pregnancy and live-birth rates, whereas ectopic pregnancy, miscarriage and neonatal outcomes were unaffected (48). Notably, cryosurvival rates of vitrified blastocysts declined markedly with longer storage (48). A 2021 study of 24,698 patients undergoing their first embryo-transfer cycle reported decreasing implantation, clinical-pregnancy, multiple-pregnancy and live-birth rates with increasing storage time, although survival rates did not differ significantly across groups (33). Similarly, Zheng et al. analyzed 6,327 vitrified blastocyst transfers and found an association between storage duration and clinical-pregnancy, ongoing-pregnancy and live-birth rates both before and after adjustment for potential confounders and propensity-score matching. The authors described a stepwise decline in reproductive outcomes, with live-birth rates of 44.57% (<3 months), 36.55% (4–6 months), 35.09% (7–12 months) and 28.77% (13–24 months) (30). Importantly, with the exception of Wang et al. (34), none of the above studies reported an impact on neonatal outcomes; that study described an increased risk of preterm birth among pregnancies derived from embryos cryostored for >12 months in a cohort of 47,006 cycles.

Several included studies reported no significant association between storage duration and reproductive outcomes. Ueno et al. (2018), in a single-center cohort of vitrified-warmed blastocysts, found no impact of cryostorage duration on pregnancy or neonatal outcomes (20). Similarly, Li et al. (2023) reported that storage duration did not affect pregnancy and neonatal outcomes after frozen-thawed embryo transfer (38), and Lee et al. (2021) found no significant differences in survival, pregnancy, or neonatal outcomes among storage-duration groups in a large single-center study (35). These null findings are important and should not be dismissed. The discrepancy between these studies and our pooled results may be explained by several factors: (1) differences in sample sizes and statistical power; (2) variations in patient populations and clinical protocols; (3) differences in the distribution of storage durations; and (4) the presence of unmeasured confounders that may vary across centers. These contradictory findings underscore the uncertainty surrounding the clinical significance of storage duration and caution against overinterpretation of our meta-analytic results.

During long-term storage, potential cryoprotectant toxicity and liquid-nitrogen contamination may exert deleterious effects on embryos (49, 50). Embryo viability could also be compromised by temperature fluctuations and radiation. Pogozhykh et al. (51) modelled repeated temperature excursions or cold-chain interruptions in biobanks by subjecting cryopreserved placental multipotent stromal cells to controlled temperature oscillations. Cell number, viability and metabolic parameters were influenced by both the number and amplitude of temperature excursions; increased apoptosis correlated only with the number of fluctuations, whereas differentiation potential remained largely unaffected. Gamma irradiation is commonly used to study the impact of storage time. Mouse embryos exposed to doses as high as 200 cGy (equivalent to 2,000 years of background radiation) showed no adverse effects on morphology, progression to morula/blastocyst, implantation or live-birth rates (52). Conversely, zebrafish embryos exposed to 1 Gy of gamma radiation exhibited increased DNA damage, reduced body length, higher mortality and more morphological anomalies (53). In clinical practice, routine procedures such as repeated opening of storage tanks or transport of specimens may affect pregnancy outcomes. It has been suggested that annual cleaning and inventory of sperm samples stored for 5–15 years could reduce post-thaw survival (54); furthermore, storage tanks with vacuum failure have higher evaporation rates than intact ones (55).

Our systematic review and meta-analysis is the most comprehensive synthesis to date on the impact of cryopreservation on embryonic competence, encompassing 17 studies. Nevertheless, our findings conflict with a previously published meta-analysis that reported no difference in clinical outcomes between early (<12 months) and long-term (>12 months) embryo transfer (56). Notably, that earlier review included only five studies and 18,047 embryos and had not incorporated the most recent evidence. In 2021, Ma et al. conducted a dose-response meta-analysis exploring the association between storage duration and pregnancy outcomes (36), including seven studies that used both vitrification and slow freezing. They concluded that storage for up to eight years had no effect on pregnancy outcomes, a conclusion derived by comparing the lowest and highest storage-duration categories within each study. We acknowledge that this interesting observation may have limited clinical utility for several reasons: (1) very few embryos were transferred beyond this duration, undermining statistical power; (2) these embryos may have been of inherently poorer morphological quality and thus lower intrinsic competence; and (3) such embryos are rarely transferred in routine IVF cycles. In contrast, we chose a 12-month cut-off because it reflects real-world clinical practice; this interval accommodates patients who need time for reproductive surgery and recovery, weight loss, correction of hormonal imbalances, interval between pregnancies, or personal, occupational or psychological considerations (57). This design ensured large sample sizes in both meta-analytic groups, enhancing statistical robustness and increasing the clinical relevance of our findings. Our results may therefore provide useful information for ART clinicians and patients when deciding whether to delay embryo transfer. Nonetheless, further studies are needed to define a precise threshold at which prognosis changes.

Several limitations must be acknowledged. First, regarding study design and causal inference: All included studies were retrospective cohorts, which are inherently susceptible to selection bias, information bias, and uncontrolled confounding. The retrospective design precludes any causal inference; our findings demonstrate association, not causation. The decision to delay embryo transfer may be correlated with patient characteristics (e.g., poor ovarian response, need for additional treatment cycles, personal circumstances, or physician discretion regarding embryo quality) that independently affect outcomes. Reverse causation is also possible: clinicians may intentionally reserve higher-quality embryos for earlier transfer and poorer-quality embryos for later use, making storage duration a proxy for embryo quality rather than an independent exposure. Second, regarding heterogeneity and its clinical sources: Substantial statistical heterogeneity was observed across all three primary outcomes (I²=78-92%), reflecting genuine clinical and methodological diversity rather than mere statistical noise. This heterogeneity stemmed from differences in: (i) patient characteristics (maternal age range 31.2-38.2 years; BMI largely unreported; infertility diagnosis and duration variably documented); (ii) embryo quality (grading systems differed across centers and were rarely adjusted for in multivariable models); (iii) laboratory practices (vitrification devices, cryoprotectant protocols, and storage tank management varied); and (iv) clinical protocols (endometrial preparation methods-natural vs. artificial cycles-were inconsistently reported, and luteal phase support protocols differed). The 95% prediction intervals for all primary outcomes crossed the null (live birth 0.88-1.61; biochemical pregnancy 0.85-2.44; clinical pregnancy 0.93-1.65), indicating that the true effect in any future clinical setting could be neutral or even harmful. Third, regarding inconsistent confounding adjustment: Critical covariates such as female age at oocyte retrieval-undoubtedly a major confounder (58)-body mass index, infertility type and duration, parity, embryo quality, developmental stage, number of embryos transferred, and endometrial preparation protocols were not consistently adjusted for across primary studies. Although our subgroup analyses stratified by confounding adjustment yielded directionally consistent results, meta-regression did not resolve the substantial heterogeneity, suggesting that residual confounding persists and likely biases effect estimates. The direction of bias is difficult to predict: if younger, healthier patients preferentially undergo earlier transfer, the observed association may be inflated; conversely, if poorer-prognosis patients are rushed to earlier transfer while better-prognosis patients delay for personal reasons, the association may be underestimated. Fourth, regarding geographic bias and generalizability: The marked predominance of Chinese studies introduces substantial geographic bias. Of 17 included studies, 12 (70.6%) were conducted in China, contributing approximately 73.3% of the statistical weight for live birth. Exclusion of Chinese studies sequentially nullified the pooled effect for all three primary outcomes (live birth: OR 1.01, 95% CI 0.82-1.23, P = 0.89). This discrepancy likely reflects: (i) younger maternal age in Chinese cohorts (mean 31.2-34.4 vs. 36-38.2 years in non-Chinese studies); (ii) more frequent double-embryo transfer policies in Chinese centers; (iii) potentially different laboratory workflows and cryostorage infrastructure; and (iv) possible publication bias favoring positive results in the Chinese literature. Crucially, non-Chinese studies (n = 4 for live birth) were individually underpowered with wide confidence intervals, and none demonstrated a significant benefit of shorter storage. These factors, rather than storage duration per se, may explain the observed geographic discrepancy. Clinicians practicing outside China should exercise particular caution in applying these findings. Fifth, regarding the dichotomous storage classification: We chose a 12-month cut-off to reflect real-world clinical practice and ensure adequate sample sizes in both groups. However, this dichotomization may mask important dose-response relationships. Our binary classification cannot distinguish between embryos stored for 13 months versus 10 years, potentially diluting or exaggerating true effects at specific durations. Future studies should employ time-to-event analyses or multiple categorical strata to better characterize dose-response patterns. Sixth, regarding long-term offspring outcomes: The absence of long-term neonatal and childhood outcome data represents a critical evidence gap and a major limitation for clinical counselling. No included study reported neurodevelopmental milestones, cognitive function, growth trajectories, metabolic health (e.g., childhood obesity, diabetes risk), cardiovascular outcomes, or childhood malignancies. The absence of such data precludes any assessment of whether prolonged cryopreservation exerts latent effects beyond the neonatal period. Parents considering extended embryo storage should be informed that current evidence provides reassurance only for perinatal outcomes, while long-term safety remains entirely unestablished. This is particularly concerning given the theoretical risks of cryoprotectant toxicity, liquid-nitrogen contamination, and cumulative radiation exposure over decades of storage.

We strongly advise against using storage duration as a standalone criterion to prioritize embryo transfer timing or to counsel patients about prognosis. The association between shorter storage and improved pregnancy rates is statistically detectable at the population level but clinically unreliable at the individual level. The effect is small, highly variable, and easily overwhelmed by well-established prognostic factors including maternal age, embryo morphological quality, endometrial receptivity, and laboratory proficiency. Decisions regarding the optimal timing of frozen-thawed embryo transfer should remain individualized, considering medical readiness, patient preferences, and embryo characteristics, rather than being driven by arbitrary storage-duration thresholds. In modern ART practice, where elective SET is the standard of care, the clinical relevance of storage duration effects may be further diminished, as the primary driver of pregnancy success shifts from quantity of embryos transferred to embryo quality and endometrial receptivity. Future research priorities should include: (1) prospective cohort studies with rigorous confounder control; (2) randomized controlled trials to establish causal effects; (3) dose-response analyses using individual patient data and flexible modeling approaches; and (4) long-term pediatric follow-up studies assessing neurodevelopmental, metabolic, and oncological outcomes in children born from embryos with varying cryostorage durations.

## Conclusion

This meta-analysis found a statistical association between shorter embryo cryopreservation duration (≤12 months) and higher rates of live birth, biochemical pregnancy, and clinical pregnancy in frozen-thawed embryo transfer cycles. However, this association does not establish causation. The higher multiple pregnancy rate observed in the ≤12-month group should be considered a potential adverse outcome, likely reflecting differences in the number of embryos transferred rather than a benefit of shorter storage. All included studies were retrospective cohorts with uncontrolled confounders, and substantial heterogeneity was present. Clinicians, particularly those practicing outside China, should exercise caution in applying these findings, as the observed associations were driven by Chinese studies and may not be generalizable to other populations or healthcare systems. The timing of transfer should be determined by individual patient circumstances, including medical readiness, personal preferences, and embryo quality, rather than by storage duration alone.

## Data Availability

The original contributions presented in the study are included in the article/**Supplementary Material**. Further inquiries can be directed to the corresponding authors.
