# Supplementary material for: Optimal timing for frozen-thawed embryo transfer: evidence from a systematic review and meta-analysis
Source: Front Endocrinol (Lausanne). 2026 Jul 15;17:1851394. doi: 10.3389/fendo.2026.1851394 (PMC13414742; doi:10.3389/fendo.2026.1851394)
Supplement: Supplementary file 1 [file SupplementaryFile1.docx]

Figure S1. Funnel plot for assessment of publication bias in live birth rate
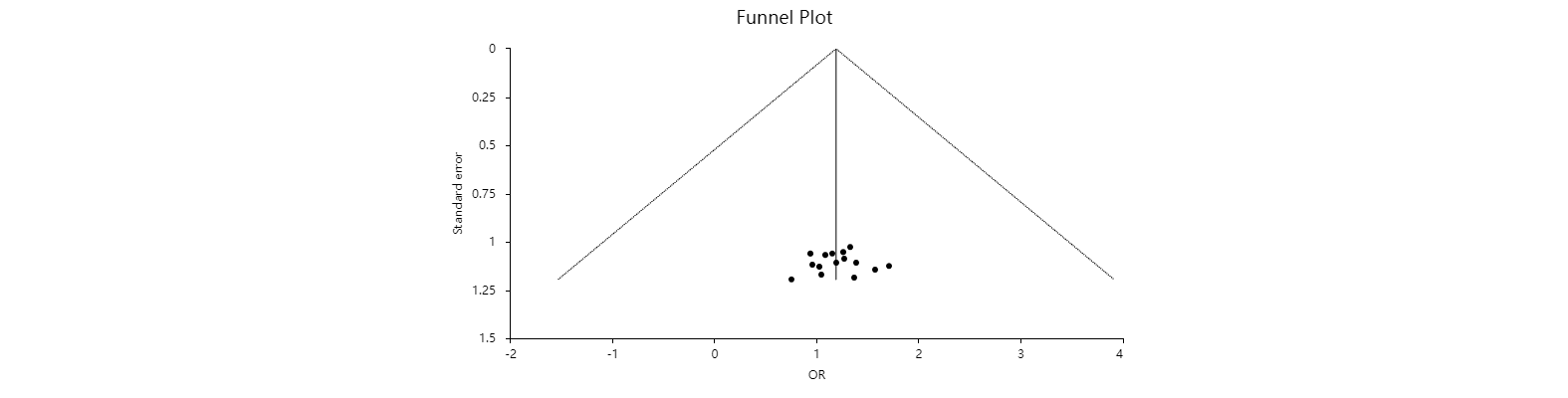
.


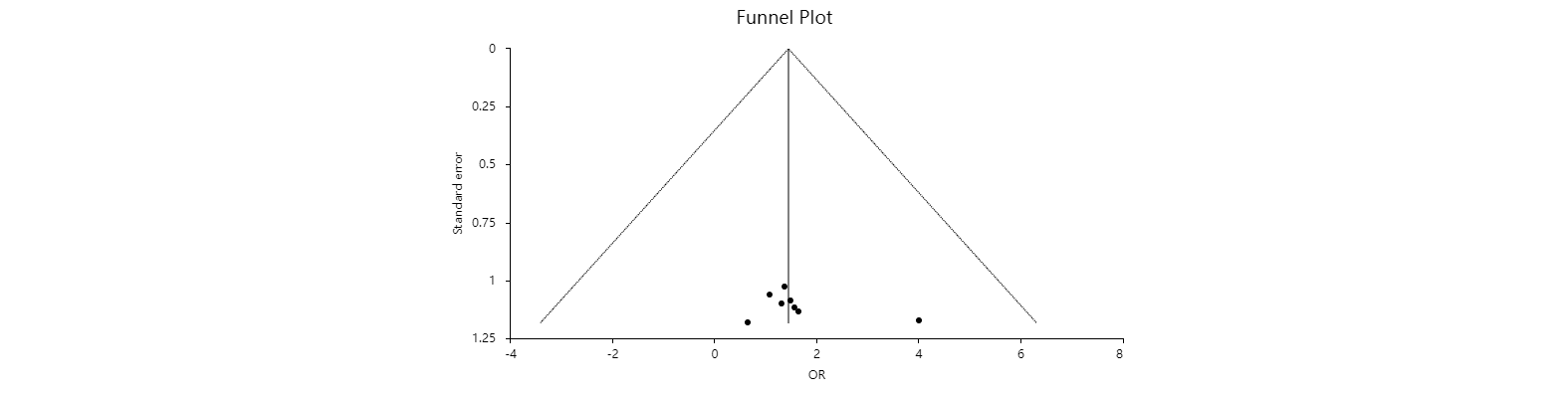


Figure S2. Funnel plot for assessment of publication bias in biochemical pregnancy rate.


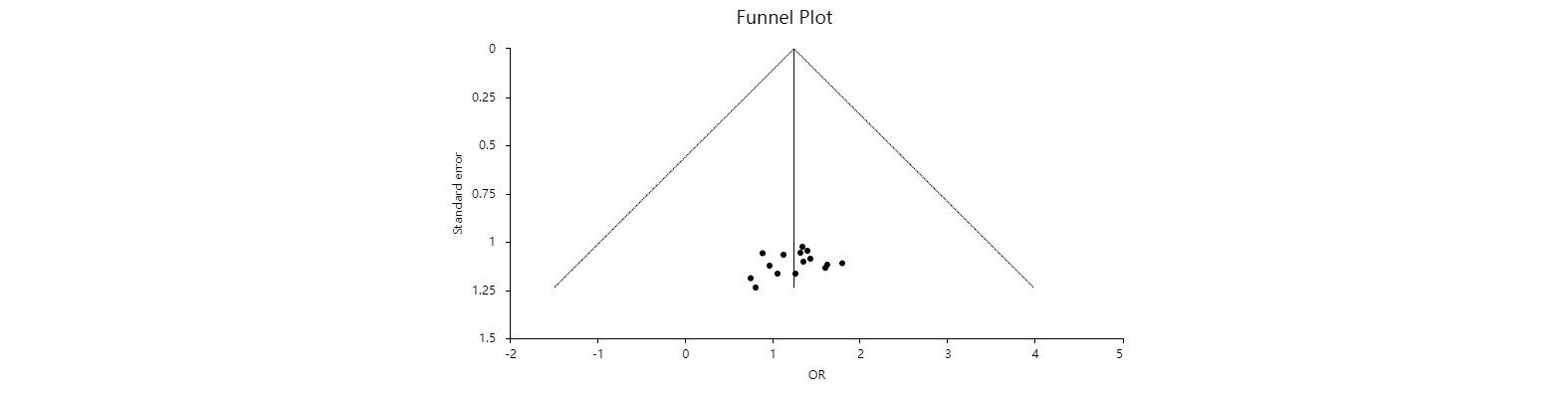


Figure S3. Funnel plot for assessment of publication bias in clinical pregnancy rate.


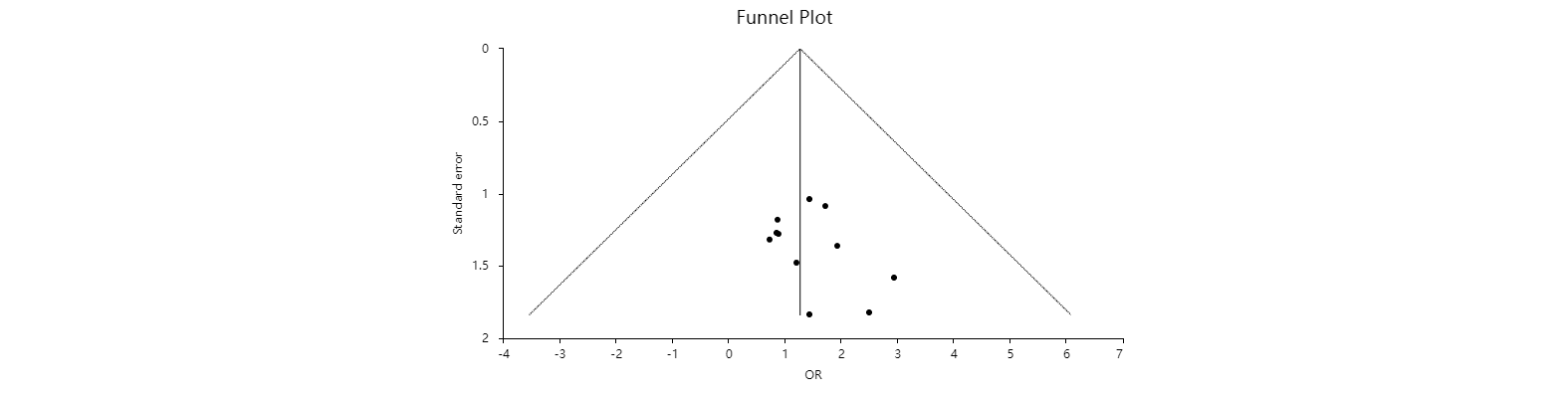


Figure S4. Funnel plot for assessment of publication bias in multiple pregnancy rate.


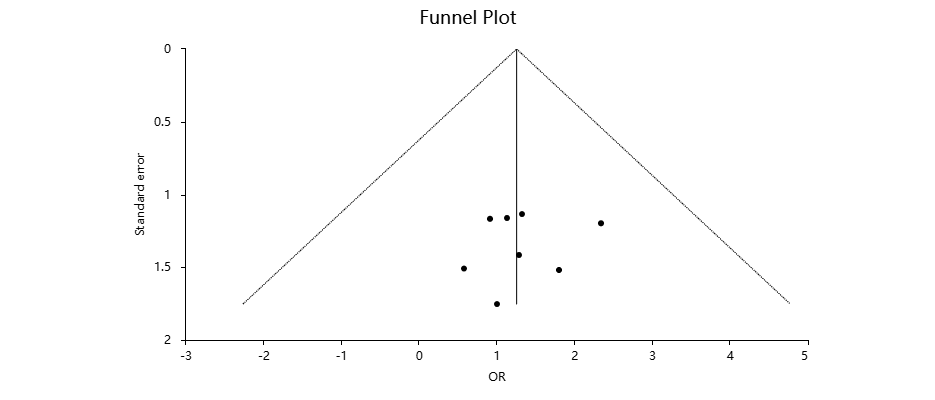


Figure S5. Funnel plot for assessment of publication bias in cryo-survival rate.


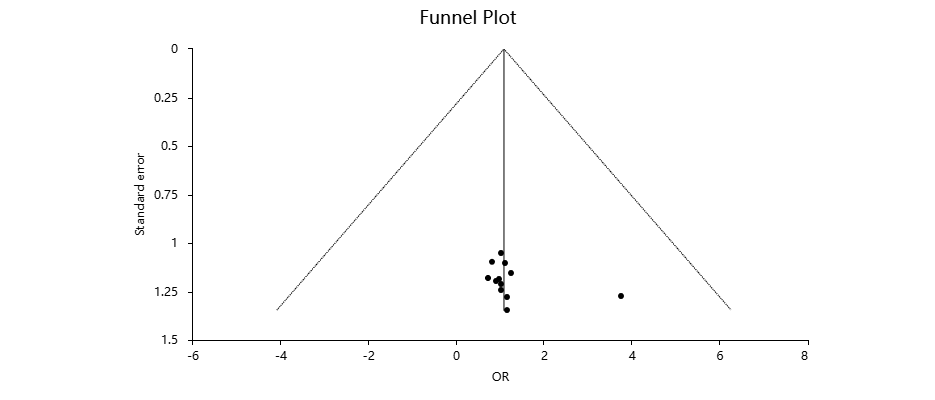


Figure S6. Funnel plot for assessment of publication bias in miscarriage rate.


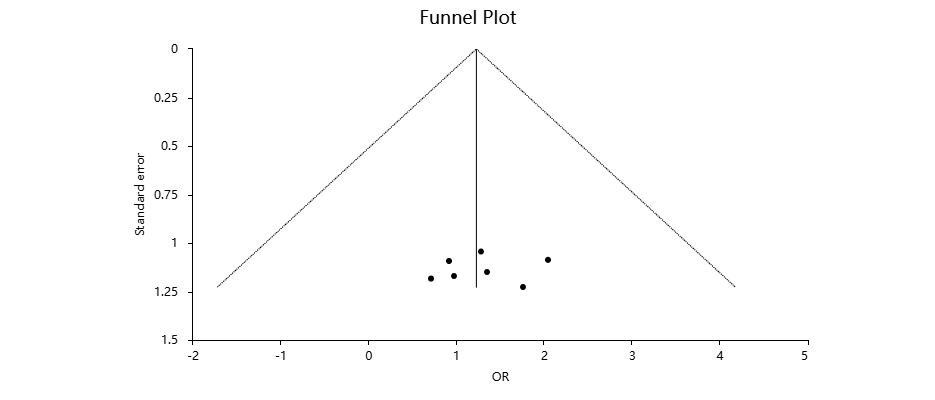


Figure S7. Funnel plot for assessment of publication bias in implantation rate.


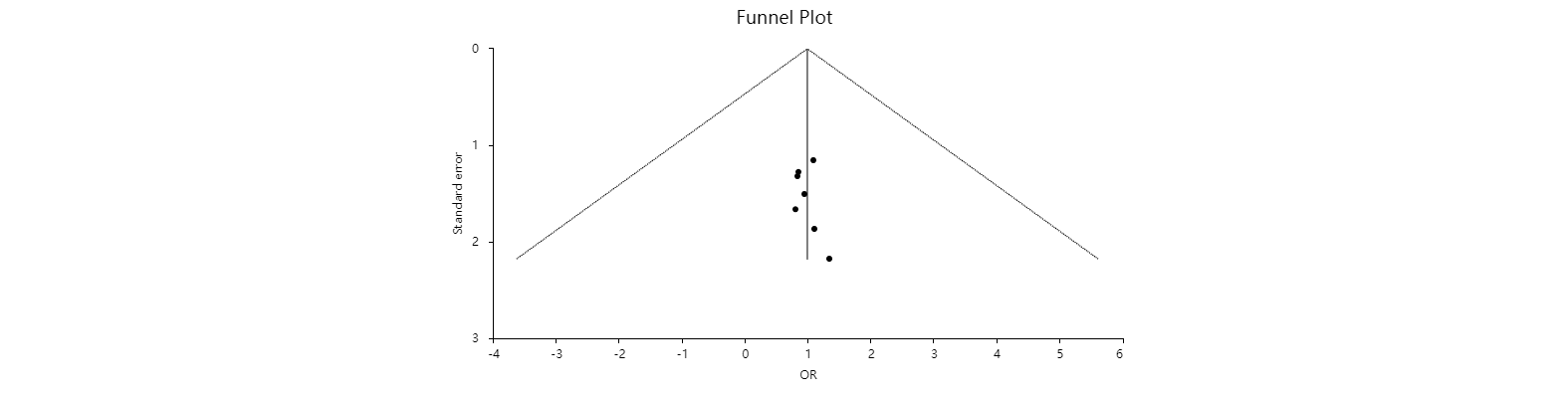


Figure S8. Funnel plot for assessment of publication bias in ectopic pregnancy rate.


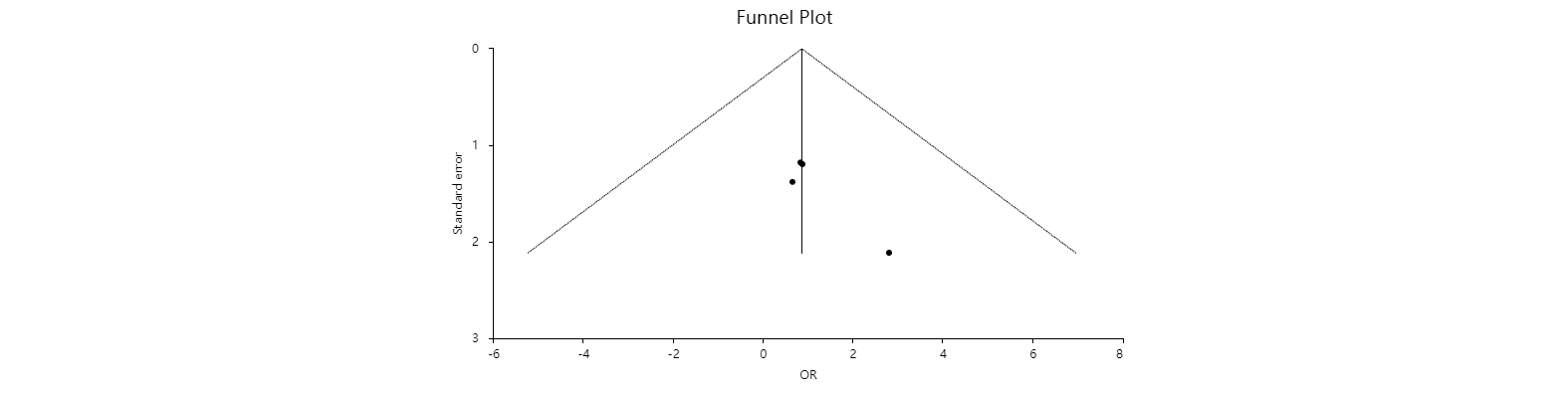


Figure S9. Funnel plot for assessment of publication bias in preterm delivery rate.


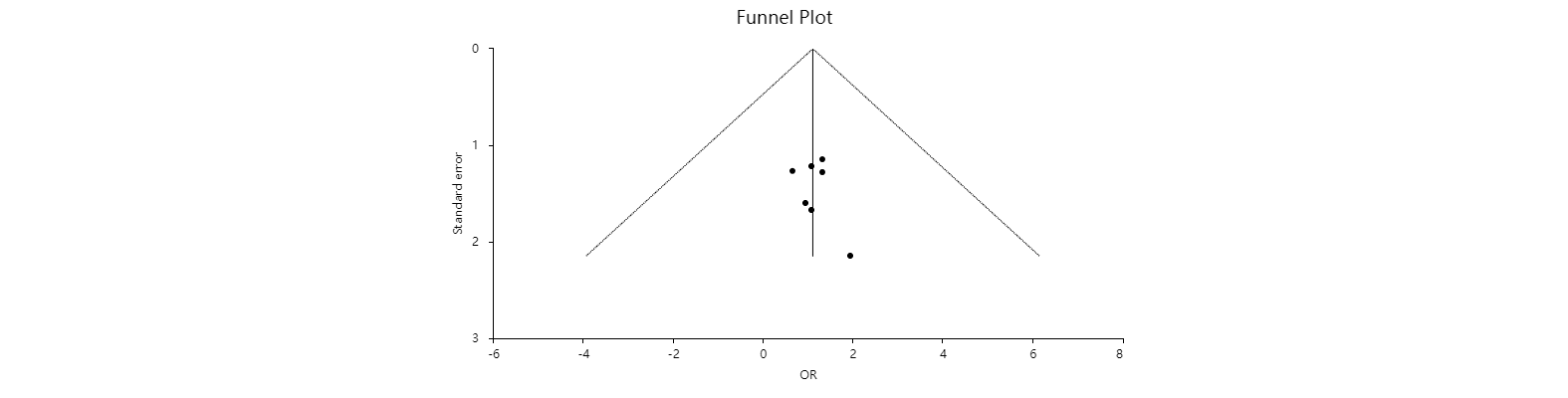


Figure S10. Funnel plot for assessment of publication bias in low birth weight.


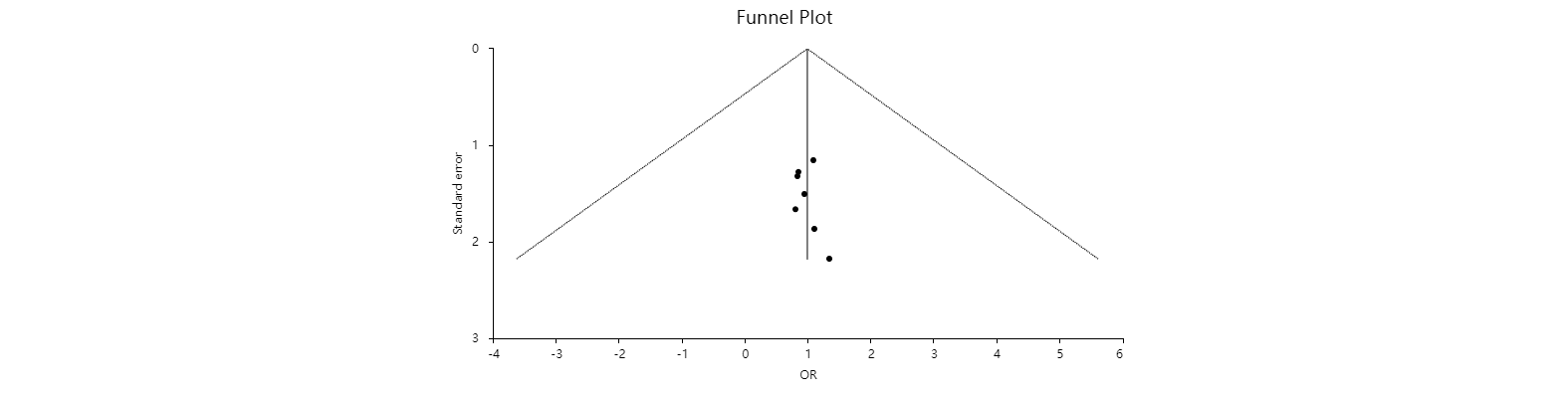


Figure S11. Funnel plot for assessment of publication bias in congenital malformation..


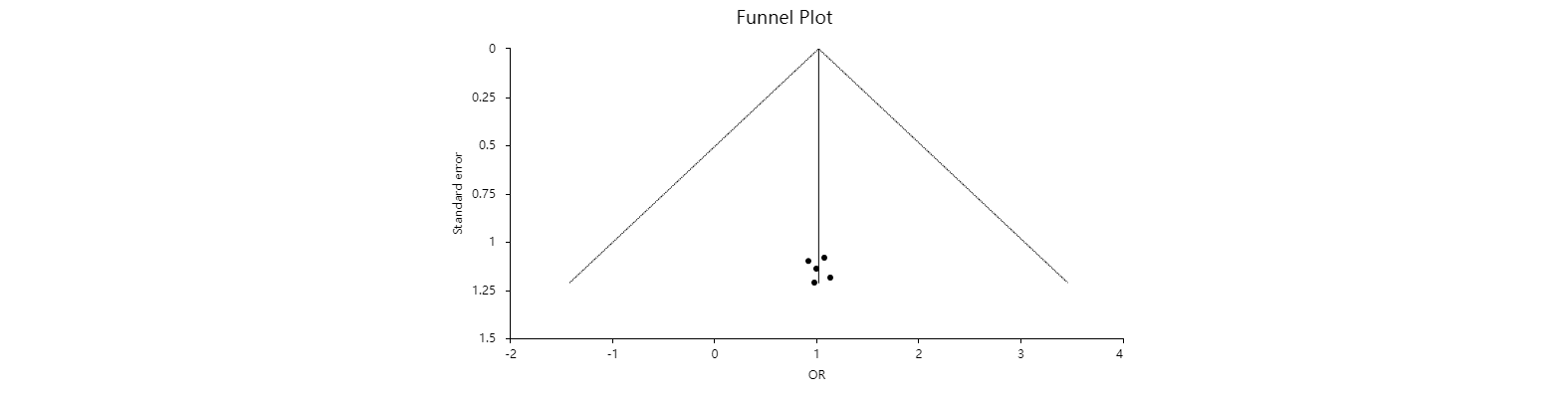


Figure S12. Funnel plot for assessment of publication bias in sex ratio.
