# Supplementary material for: Optimal timing for frozen-thawed embryo transfer: evidence from a systematic review and meta-analysis
Source: Front Endocrinol (Lausanne). 2026 Jul 15;17:1851394. doi: 10.3389/fendo.2026.1851394 (PMC13414742; doi:10.3389/fendo.2026.1851394)
Supplement: Supplementary file 2 [file SupplementaryFile2.pdf]

Table S1. Leave-one-out sensitivity analysis for live birth rate.

| Study | Adjusted OR (95% CI) | P value | $I^2$(%) |
|---|---|---|---|
| (-)Cimadomo | 1.21 [1.10, 1.32] | Z = 4.15 (P < 0.0001) | 77.74 |
| (-)Cui | 1.22 [1.13, 1.32] | Z = 4.89 (P < 0.00001) | 68.3 |
| (-)Hu | 1.17 [1.07, 1.28] | Z = 3.48 (P = 0.0005) | 78.33 |
| (-)Lee | 1.20 [1.10, 1.31] | Z = 3.97 (P < 0.0001) | 78.73 |
| (-)LiW | 1.18 [1.08, 1.29] | Z = 3.64 (P = 0.0003) | 79.37 |
| (-)LiX. | 1.20 [1.09, 1.31] | Z = 3.91 (P < 0.0001) | 79.15 |
| (-)Liang | 1.18 [1.07, 1.29] | Z = 3.48 (P = 0.0005) | 79.04 |
| (-)Lin | 1.18 [1.07, 1.30] | Z = 3.35 (P = 0.0008) | 79.40 |
| (-)Ma | 1.19 [1.08, 1.30] | Z = 3.67 (P = 0.0002) | 79.48 |
| (-)Mao | 1.19 [1.08, 1.31] | Z = 3.60 (P = 0.0003) | 79.07 |
| (-)Ueno | 1.20 [1.09, 1.31] | Z = 3.83 (P = 0.0001) | 78.17 |
| (-)Wang | 1.17 [1.07, 1.29] | Z = 3.36 (P = 0.0008) | 72.46 |
| (-)Wireitner | 1.21 [1.11, 1.32] | Z = 4.39 (P < 0.0001) | 76.81 |
| (-)Zhang | 1.16 [1.07, 1.27] | Z = 3.43 (P = 0.0006) | 76.56 |
| (-)Zheng | 1.18 [1.08, 1.30] | Z = 3.53 (P = 0.0004) | 79.46 |
| Pooled effect | 1.19 [1.09, 1.30] | Z = 3.89 (P < 0.0001) | 77.92 |

.

Table S2. Leave-one-out sensitivity analysis for biochemical pregnancy rate.

| **Study** | **Adjusted OR (95% CI)** | **P value** | **$I^2$(%)** |
|---|---|---|---|
| (-)Cui | 1.51 [1.21, 1.89] | z = 3.64, p = 0.0003 | 91.04 |
| (-)Hu | 1.42 [1.15, 1.75] | z = 3.25, p = 0.001 | 92.63 |
| (-)Li X | 1.29 [1.12, 1.49] | z = 3.49, p = 0.0005 | 84.05 |
| (-)Liang | 1.47 [1.18, 1.83] | z = 3.42, p = 0.0006 | 92.84 |
| (-)Wang | 1.46 [1.10, 1.94] | z = 2.63, p = 0.009 | 92.81 |
| (-)Wirleitner | 1.57 [1.30, 1.89] | z = 4.78, p < 0.00001 | 90.81 |
| (-)Zhang | 1.43 [1.15, 1.77] | z = 3.26, p = 0.001 | 92.71 |
| (-)Zheng | 1.44 [1.15, 1.80] | z = 3.19, p = 0.001 | 92.75 |
| Pooled effect | 1.44 [1.19, 1.75] | z = 3.74, p = 0.0002 | 91.66 |

Table S3. Leave-one-out sensitivity analysis for clinical pregnancy rate.

| Study | Adjusted OR (95% CI) | P value | $I^2$(%) |
|---|---|---|---|
| (-)Aflatoonian | 1.26 [1.14, 1.39] | Z = 4.45 (P < 0.00001) | 85.66 |
| (-)Cui | 1.29 [1.19, 1.40] | Z = 6.00 (P < 0.00001) | 73.83 |
| (-)Hu | 1.22 [1.10, 1.35] | Z = 3.73 (P = 0.0002) | 85.91 |
| (-)Lee | 1.26 [1.14, 1.40] | Z = 4.41 (P < 0.0001) | 85.42 |
| (-)LiW | 1.24 [1.11, 1.37] | Z = 3.97 (P < 0.0001) | 86.37 |
| (-)LiX. | 1.25 [1.13, 1.39] | Z = 4.20 (P < 0.0001) | 86.13 |
| (-)Liang | 1.23 [1.11, 1.37] | Z = 3.81 (P = 0.0001) | 86.34 |
| (-)Lin | 1.22 [1.09, 1.37] | Z = 3.48 (P = 0.0005) | 85.71 |
| (-)Ma | 1.21 [1.09, 1.34] | Z = 3.66 (P = 0.0003) | 84.65 |
| (-)Mao | 1.23 [1.10, 1.38] | Z = 3.61 (P = 0.0003) | 86.33 |
| (-)Ueno | 1.25 [1.12, 1.39] | Z = 4.03 (P < 0.0001) | 85.68 |
| (-)Wang | 1.22 [1.08, 1.38] | Z = 3.25 (P = 0.001) | 85.62 |
| (-)Wirleitner | 1.27 [1.15, 1.40] | Z = 4.70 (P < 0.00001) | 84.82 |
| (-)Zhang | 1.22 [1.10, 1.35] | Z = 3.69 (P = 0.0002) | 85.7 |
| (-)Zheng | 1.22 [1.10, 1.36] | Z = 3.70 (P = 0.0002) | 86.11 |
| Pooled effect | 1.24 [1.12, 1.37] | Z = 4.16 (P < 0.0001) | 85.33 |

Table S4. Leave-one-out sensitivity analysis for multiple pregnancy rate.

| **Study** | **Adjusted OR (95% CI)** | **P value** | **$I^2$(%)** |
|---|---|---|---|
| (-)Cui | 1.31 [1.07, 1.62] | Z = 2.55 (P = 0.01) | 68.50 |
| (-)Lee | 1.35 [1.10, 1.64] | Z = 2.92 (P = 0.003) | 61.42 |
| (-)LiW | 1.27 [1.02, 1.57] | Z = 2.18 (P = 0.03) | 71.98 |
| (-)LiX. | 1.32 [1.07, 1.62] | Z = 2.62 (P = 0.009) | 67.69 |
| (-)Liang | 1.22 [0.99, 1.51] | Z = 1.85 (P = 0.06) | 71.29 |
| (-)Lin | 1.33 [1.09, 1.62] | Z = 2.82 (P = 0.005) | 65.72 |
| (-)Ma | 1.24 [1.01, 1.53] | Z = 2.05 (P = 0.04) | 71.34 |
| (-)Mao | 1.19 [0.92, 1.54] | Z = 1.34 (P = 0.18) | 65.72 |
| (-)Ueno | 1.26 [1.02, 1.55] | Z = 2.17 (P = 0.03) | 72.14 |
| (-)Wang | 1.24 [0.92, 1.68] | Z = 1.42 (P = 0.16) | 71.92 |
| (-)Wirleitner | 1.22 [1.00, 1.50] | Z = 1.93 (P = 0.05) | 69.81 |
| Pooled effect | 1.26 [1.03, 1.55] | Z = 2.27 (P = 0.02) | 69.04 |

Table S5. Leave-one-out sensitivity analysis for cryo-survival rate.

| Study | Adjusted OR (95% CI) | P value | $I^2$(%) |
|---|---|---|---|
| (-)Cimadomo | 1.21 [0.90, 1.63] | Z = 1.29 (P = 0.20) | 70.92 |
| (-)Cui | 1.25 [0.92, 1.69] | Z = 1.41 (P = 0.16) | 71.91 |
| (-)Lee | 1.27 [0.90, 1.79] | Z = 1.37 (P = 0.17) | 71.08 |
| (-)LiW | 1.33 [1.00, 1.75] | Z = 2.07 (P = 0.05) | 65.82 |
| (-)Lin | 1.34 [0.98, 1.81] | Z = 1.94 (P = 0.06) | 61.63 |
| (-)Ma | 1.27 [0.94, 1.70] | Z = 1.58 (P = 0.11) | 71.68 |
| (-)Ueno | 1.12 [0.93, 1.35] | Z = 1.23 (P = 0.22) | 21.24 |
| (-)Wirleitner | 1.23 [0.86, 1.76] | Z = 1.11 (P = 0.27) | 71.76 |
| Pooled effect | 1.25 [0.95, 1.65] | Z = 1.59 (P = 0.11) | 67.23 |

Table S6. Leave-one-out sensitivity analysis for miscarriage rate.

| **Study** | **Adjusted OR (95% CI)** | **P value** | **$I^2$(%)** |
|---|---|---|---|
| (-)Cmadomo | 0.99 [0.90, 1.08] | Z = 0.26 (P = 0.80) | 25.87 |
| (-)Cui | 1.12 [0.95, 1.32] | Z = 1.33 (P = 0.18) | 74.72 |
| (-)Hu | 1.08 [0.91, 1.27] | Z = 0.84 (P = 0.40) | 77.15 |
| (-)Lee | 1.09 [0.92, 1.29] | Z = 0.94 (P = 0.35) | 77.3 |
| (-)LiX. | 1.08 [0.91, 1.27] | Z = 0.85 (P = 0.39) | 77.18 |
| (-)Liang | 1.09 [0.92, 1.30] | Z = 0.98 (P = 0.33) | 77.28 |
| (-)Lin | 1.12 [0.94, 1.33] | Z = 1.28 (P = 0.20) | 72.94 |
| (-)Ma | 1.10 [0.92, 1.30] | Z = 1.05 (P = 0.29) | 77.10 |
| (-)Ueno | 1.08 [0.90, 1.30] | Z = 0.84 (P = 0.40) | 76.89 |
| (-)Wang | 1.10 [0.90, 1.35] | Z = 0.91 (P = 0.36) | 77.40 |
| (-)Zhang | 1.09 [0.91, 1.29] | Z = 0.94 (P = 0.35) | 77.30 |
| (-)Zheng | 1.06 [0.90, 1.26] | Z = 0.70 (P = 0.48) | 76.08 |
| Pooled effect | 1.08 [0.92, 1.27] | Z = 0.93 (P = 0.35) | 75.02 |

Table S7. Leave-one-out sensitivity analysis for implantation rate.

| Study | Adjusted OR (95% CI) | P value | $I^2$(%) |
|---|---|---|---|
| (-)Aflatoonian | 1.17 [0.90, 1.52] | Z = 1.15 (P = 0.25) | 91.69 |
| (-)Lee | 1.29 [0.99, 1.68] | Z = 1.90 (P = 0.06) | 89.22 |
| (-)LiW. | 1.21 [0.91, 1.59] | Z = 1.31 (P = 0.19) | 92.00 |
| (-)LiX. | 1.27 [0.97, 1.66] | Z = 1.74 (P = 0.08) | 91.57 |
| (-)Mao | 1.21 [0.85, 1.73] | Z = 1.05 (P = 0.29) | 92.01 |
| (-)Wirleitner | 1.33 [1.04, 1.70] | Z = 2.25 (P = 0.02) | 90.03 |
| (-)Zheng | 1.11 [0.90, 1.37] | Z = 1.00 (P = 0.32) | 81.15 |
| Pooled effect | 1.23 [0.96, 1.56] | Z = 1.63 (P = 0.10) | 90.42 |

Table S8. Leave-one-out sensitivity analysis for ectopic pregnancy rate.

| **Study** | **Adjusted OR (95% CI)** | **P value** | **$I^2$(%)** |
|---|---|---|---|
| (-)Cui | 0.99 [0.81, 1.22] | Z = 0.07 (P = 0.94) | 0 |
| (-)LiX. | 0.98 [0.80, 1.20] | Z = 0.16 (P = 0.87) | 0 |
| (-)Lin | 1.02 [0.82, 1.27] | Z = 0.18 (P = 0.86) | 0 |
| (-)Ma | 0.99 [0.80, 1.21] | Z = 0.14 (P = 0.89) | 0 |
| (-)Mao | 1.01 [0.82, 1.26] | Z = 0.12 (P = 0.91) | 0 |
| (-)Wang | 0.89 [0.66, 1.19] | Z = 0.81 (P = 0.42) | 0 |
| (-)Zhang | 1.00 [0.81, 1.22] | Z = 0.03 (P = 0.98) | 0 |
| Pooled effect | 0.99 [0.81, 1.21] | Z = 0.11 (P = 0.91) | 0 |

Table S9. Leave-one-out sensitivity analysis for preterm delivery rate.

| **Study** | **Adjusted OR (95% CI)** | **P value** | **$I^2$(%)** |
|---|---|---|---|
| (-)Cui | 0.90 [0.66, 1.22] | Z = 0.69 (P = 0.49) | 39.45 |
| (-)Lee | 0.86 [0.65, 1.14] | Z = 1.04 (P = 0.30) | 38.47 |
| (-)Li J. | 0.90 [0.71, 1.14] | Z = 0.86 (P = 0.39) | 19.49 |
| (-)LiX. | 0.83 [0.67, 1.05] | Z = 1.58 (P = 0.12) | 0 |
| Pooled effect | 0.87 [0.70, 1.09] | Z = 1.23 (P = 0.22) | 9.08 |

Table S10. Leave-one-out sensitivity analysis for low birth weight.

| **Study** | **Adjusted OR (95% CI)** | **P value** | **$I^2$(%)** |
| --- | --- | --- | --- |
| (-)Cimadomo | 1.25 [0.98, 1.51] | Z = 1.77 (P = 0.11) | 0 |
| (-)Cui | 1.12 [0.93, 1.35] | Z = 1.21 (P = 0.23) | 33.45 |
| (-)Lee | 1.16 [0.96, 1.41] | Z = 1.50 (P = 0.13) | 36.87 |
| (-)LiJ. | 1.15 [0.96, 1.37] | Z = 1.54 (P = 0.12) | 37.11 |
| (-)Li X. | 1.14 [0.95, 1.36] | Z = 1.43 (P = 0.15) | 32.88 |
| (-)Liang | 1.16 [0.97, 1.38] | Z = 1.59 (P = 0.11) | 35.99 |
| (-)Mao | 1.02 [0.81, 1.29] | Z = 0.18 (P = 0.86) | 10.76 |
| Pooled effect | 1.15 [0.96, 1.37] | Z = 1.54 (P = 0.12) | 24.59 |

Table S11. Leave-one-out sensitivity analysis for congenital malformation.

| Study | Adjusted OR (95% CI) | P value | $I^2$(%) |
|---|---|---|---|
| (-)Cimadomo | 0.88 [0.63, 1.21] | Z = 0.80 (P = 0.42) | 0 |
| (-)Cui | 0.98 [0.67, 1.42] | Z = 0.11 (P = 0.91) | 11.38 |
| (-)Lee | 0.97 [0.70, 1.33] | Z = 0.22 (P = 0.83) | 0 |
| (-)Li J. | 0.93 [0.68, 1.27] | Z = 0.45 (P = 0.65) | 11.63 |
| (-)Li W. | 0.93 [0.68, 1.28] | Z = 0.43 (P = 0.67) | 12.12 |
| (-)Lin | 1.00 [0.72, 1.40] | Z = 0.02 (P = 0.98) | 0 |
| (-)Ma | 0.88 [0.65, 1.21] | Z = 0.77 (P = 0.44) | 0 |
| (-)Mao | 0.93 [0.65, 1.31] | Z = 0.44 (P = 0.66) | 11.82 |
| (-)Ueno | 0.89 [0.63, 1.25] | Z = 0.67 (P = 0.50) | 6.47 |
| (-)Wirleitner | 0.98 [0.71, 1.35] | Z = 0.12 (P = 0.90) | 0 |
| Pooled effect | 0.94 [0.69, 1.28] | Z = 0.42 (P = 0.67) | 1.3 |

Table S12. Leave-one-out sensitivity analysis for sex ratio.

| **Study** | **Adjusted OR (95% CI)** | **P value** | **$I^2$(%)** |
|---|---|---|---|
| (-)Cui | 1.06 [0.94, 1.19] | Z = 0.95 (P = 0.34) | - |
| (-)Lee | 1.01 [0.91, 1.12] | Z = 0.13 (P = 0.90) | 13.574 |
| (-)Li J. | 1.02 [0.92, 1.13] | Z = 0.40 (P = 0.69) | 0 |
| (-)Mao | 0.98 [0.86, 1.11] | Z = 0.32 (P = 0.75) | 0 |
| (-)Wang | 1.02 [0.92, 1.14] | Z = 0.38 (P = 0.70) | 0 |
| Pooled effect | 1.02 [0.92, 1.12] | Z = 0.35 (P = 0.72) | 0 |

Table S13. Sensitivity analyses beyond Leave-one-out

| Analysis type | Stratum | Studies (n) | Pooled OR [95% CI] | $I^2$ (%) | P for interaction |
|---|---|---|---|---|---|
| Live birth rate | | | | | |
| Study quality (NOS) | ≤6 (moderate) | 6 | 1.15 [0.98, 1.35] | 72.3 | 0.42 |
| | ≥7 (high) | 9 | 1.21 [1.08, 1.36] | 81.5 | |
| Confounding adjustment | Unadjusted | 5 | 1.12 [0.53, 2.37] | 74.9 | 0.72 |
| | Adjusted | 10 | 1.20 [1.07, 1.34] | 80.2 | |
| Embryo transfer policy | SET only | 6 | 1.14 [0.96, 1.36] | 78.1 | 0.38 |
| | SET/DET mixed | 9 | 1.22 [1.09, 1.38] | 78.6 | |
| Country | China | 11 | 1.26 [1.13, 1.41] | 76.95 | <0.001 |
| | Other countries | 4 | 1.01 [0.82, 1.23] | 23.73 | |
| Biochemical pregnancy rate | | | | | |
| Study quality (NOS) | ≤6 (moderate) | 2 | 1.38 [0.89, 2.14] | 93.1 | 0.55 |
| | ≥7 (high) | 6 | 1.51 [1.15, 1.98] | 90.4 | |
| Confounding adjustment | Unadjusted | 2 | 0.66 [0.48, 0.92] | 0 | <0.001 |
| | Adjusted | 6 | 1.57 [1.10, 2.25] | 90.8 | |
| Embryo transfer policy | SET only | 2 | 1.35 [0.78, 2.34] | 93.5 | 0.67 |
| | SET/DET mixed | 6 | 1.52 [1.11, 2.08] | 91.2 | |
| Country | China | 7 | 1.57 [1.10, 2.25] | 90.81 | <0.001 |
| | Other countries | 1 | 0.66 [0.48, 0.92] | 0.00 | |
| Clinical pregnancy rate | | | | | |
| Study quality (NOS) | ≤6 (moderate) | 6 | 1.19 [1.01, 1.41] | 83.2 | 0.31 |
| | ≥7 (high) | 9 | 1.27 [1.11, 1.46] | 87.1 | |
| Confounding adjustment | Unadjusted | 6 | 1.05 [0.64, 1.74] | 83.1 | 0.25 |
| | Adjusted | 9 | 1.28 [1.10, 1.49] | 87.0 | |
| Embryo transfer policy | SET only | 5 | 1.18 [0.98, 1.42] | 85.4 | 0.29 |
| | SET/DET mixed | 10 | 1.28 [1.12, 1.46] | 85.2 | |
| Country | China | 11 | 1.34 (1.17-1.53) | 85.37 | <0.001 |
| | Other countries | 4 | 0.95 (0.71-1.28) | 57.26 | |

Note. SET, single-embryo transfer; DET, double-embryo transfer; NOS, Newcastle-Ottawa Scale. For biochemical pregnancy rate, analyses were limited by small stratum sizes; results should be interpreted with caution.
